# Association between phthalates exposure and non-alcoholic fatty liver disease under different diagnostic criteria: a cross-sectional study based on NHANES 2017 to 2018

**DOI:** 10.3389/fpubh.2024.1407976

**Published:** 2024-09-25

**Authors:** Jiazhen Zou, Qingdan Gu, Dayong Gu

**Affiliations:** ^1^Department of Laboratory Medicine, Shenzhen Institute of Translational Medicine, The First Affiliated Hospital of Shenzhen University, Shenzhen Second People’s Hospital, Shenzhen Key Laboratory of Medical Laboratory and Molecular Diagnostics, Shenzhen, China; ^2^Shenzhen Yantian District People’s Hospital (Group), Southern University of Science and Technology Yantian Hospital, Shenzhen, China

**Keywords:** NAFLD, phthalates, National Health and Nutrition Examination Survey (NHANES), MCiOP, DINP

## Abstract

**Purpose:**

Non-alcoholic fatty liver disease (NAFLD) is the most common liver disease. Phthalates have been suggested to influence the development of NAFLD due to their endocrine-disrupting properties, but studies based on nationally representative populations are insufficient, and existing studies seem to have reached conflicting conclusions. Due to changes in legislation, the use of traditional phthalates has gradually decreased, and the phthalates substitutes is getting more attention. This study aims to delve deeper into how the choice of diagnostic approach influences observed correlations and concern about more alternatives of phthalates, thereby offering more precise references for the prevention and treatment of NAFLD.

**Methods:**

A cohort of 641 participants, sourced from the National Health and Nutrition Examination Survey (NHANES) 2017–2018 database, was evaluated for NAFLD using three diagnostic methods: the Hepatic Steatosis Index (HSI), the US Fatty Liver Indicator (US.FLI), and Vibration Controlled Transient Elastography (VCTE). The urinary metabolite concentrations of Di-2-ethylhexyl phthalate (DEHP), Di-isodecyl phthalate (DIDP), Di-isononyl phthalate (DINP), Di-n-butyl phthalate (DnBP), Di-isobutyl phthalate (DIBP), Di-ethyl phthalate (DEP) and Di-n-octyl phthalate (DnOP) were detected. The association between NAFLD and urinary phthalate metabolites was evaluated through univariate and multivariate logistic regression analyses, considering different concentration gradients of urinary phthalates.

**Results:**

Univariate logistic regression analysis found significant correlations between NAFLD and specific urinary phthalate metabolites, such as Mono-(2-ethyl-5-oxohexyl) phthalate (MEOHP), Mono-(2-ethyl-5-hydroxyhexyl) phthalate (MEHHP), Mono-2-ethyl-5-carboxypentyl phthalate (MECPP), and Mono-(carboxyisoctyl) phthalate (MCiOP), across different diagnostic criteria. In a multivariate logistic regression analysis adjusting only for demographic data, MEOHP (OR = 3.26, 95% CI = 1.19–8.94, *p* = 0.029), MEHHP (OR = 3.98, 95% CI = 1.43–11.1, *p* = 0.016), MECPP (OR = 3.52, 95% CI = 1.01–12.2, *p* = 0.049), and MCiOP (OR = 4.55, 95% CI = 1.93–10.7, *p* = 0.005) were positively related to NAFLD defined by HSI and VCTE. The correlation strength varied with the concentration of phthalates, indicating a potential dose–response relationship. Adjusting for all covariates in multivariate logistic regression, only MCiOP (OR = 4.22, 95% CI = 1.10–16.2, *p* = 0.044), as an oxidative metabolite of DINP, remained significantly associated with NAFLD under the VCTE criterion, suggesting its potential role as a risk factor for NAFLD.

**Conclusion:**

This research highlights a significant association between DINP and NAFLD. These findings underscore the need for further investigation into the role of the phthalates substitutes in the pathogenesis of NAFLD and the importance of considering different diagnostic criteria in research.

## 1 Introduction

Non-alcoholic fatty liver disease (NAFLD) is the most common cause of chronic liver disease and is likely to be the most common indication for liver transplantation by 2030 (1). A meta-analysis of studies published between 1990 and 2015 suggests that the global prevalence of NAFLD is about 25% (2). As of 2019, the global total prevalence was as high as 30.05%, which increased by about 50 percent between 2016–2019, affecting 37% of Americans (3, 4). NAFLD is a liver disease associated with a metabolic disorder characterized by the accumulation of excessive fat in the liver, a condition that is not caused by alcohol consumption (5). The progression of the disease includes steatohepatitis, fibrosis, and eventually, possibly even cirrhosis (6). Multiple factors play an important role in the disease: insulin resistance (7), lipid accumulation, oxidative stress (8), gene mutation (5), sedentary lifestyle and eating patterns (6). In recent years, the relationship between phthalates exposure and diseases has been concerned.

Phthalates, widely used plasticizers in PVC and various plastics, confer flexibility and durability but raise health concerns due to their endocrine-disrupting potential and environmental pervasiveness (9). In daily life, people inevitably encounter phthalates and their metabolites through personal care products, detergents, food packaging, and even some medical devices (10). Ingestion and inhalation are two important routes for phthalates to enter the body, and intake is most often assessed by measuring metabolites in urine (11). Upon entering the human body, phthalates undergo extensive metabolic processing, yielding a multitude of metabolites. We summarize the relationships between the metabolites and their parent materials, as well as their chemical formulas, for the reader’s reference (Supplementary Table S1; Supplementary Figure S1). Phthalates have been linked to a variety of diseases, such as hyperuricemia (12), reproductive health (13), cardiovascular disease risk (14, 15), metabolic syndrome (16) and liver injury (17).

The studies that have been published so far have applied different diagnostic methods to reach controversial conclusions. A study based on the database of a representative population in Korea, The Korean National Environmental Health Survey (KoNEHS), indicated that the higher concentration of MEHHP were positively correlated with the prevalence of NAFLD using HIS diagnostic criteria (18). However, the results of another study based on the US population showed that not only MEHHP, but also MEOHP and MECPP showed a significant positive association with NAFLD employing the HSI diagnostic criteria. Unfortunately, only MEHHP maintained this correlation when they were based on the US.FLI standard (19). Furthermore, a recent study found that under the diagnostic criteria of HSI and US.FLI, there is no correlation between MEHHP, MEOHP, and MECPP with NAFLD. However, based on the VCTE standard, MEHHP is positively correlated with NAFLD (20). Another similar study also employed the VCTE diagnostic criteria, and they found that, after adjusting for all variables, the above three metabolites are not associated with NAFLD (20, 21). Clearly, different researchers applying various diagnostic criteria to assess NAFLD have reached controversial conclusions. Currently, there are no studies that simultaneously apply all three criteria to the same group of subjects for diagnosis and compare how the choice of diagnostic methods affects the evaluation of disease relevance.

Due to changes in legislation, the use of DEHP has gradually decreased, and the phthalates substitutes is getting more attention. For metabolites of DEHP alternatives, MEP can elevate levels of alanine aminotransferase (ALT) and aspartate aminotransferase (AST), enzymes potentially indicative of liver disease (22). Within the European Union (EU), DEHP is recognized as harmful to reproduction and requires clear labeling with a skull-and-crossbones symbol and the word “TOXIC” when in pure form (Regulation of DEHP | Health Care Without Harm^1^). For drugs and biological products under the CDER’s jurisdiction, the FDA strongly discourages the use of DEHP as an excipient (FDA-2012-D-1135). As a result of these regulations, the use of DEHP is decreasing, but the use of its alternatives is gradually increasing, such as DINP (23). In addition, other DEHP alternatives are also worthy of attention, such as DIDP, DINP, DnBP, DIBP, DEP and DnOP. MnBP, a metabolite of DnBP, is believed to be correlated with NAFLD (24). However, the research on DEHP substitutes and liver diseases is still insufficient, and the research on the correlation of alternative compounds and NAFLD is even less known.

This study utilized nationally representative data from the National Health and Nutrition Examination Surveys (NHANES) database in the United States to explore the association between phthalates and NAFLD under different prediction criteria (including HSI, US.FLI and VCTE). It is recommended that healthcare professionals closely examine the association between different metabolites and disease when selecting predictive models other than the gold standard for evaluating NAFLD, to enhance the accuracy of preventive measures.

## 2 Materials and methods

### 2.1 Study design and participants

The National Health and Nutrition Examination Survey (NHANES) is a significant public health database to assess the health and nutritional status of adults and children in the United States. It’s conducted by the National Center for Health Statistics (NCHS), which is part of the Centers for Disease Control and Prevention (CDC). In the process of implementation, researchers conduct laboratory and imaging tests in specially designed and equipped Mobile Examination Centers (MECs). The use of professional collection, processing, and storage protocols ensures that the data obtained from the NHANES database is both accurate and reliable. The analysis of this data not only represents the specific population sampled, but also considering the database employs a complex, multistage probability sampling design to select participants, it is representative of the broader civilian U.S. population.

**Figure 1:**
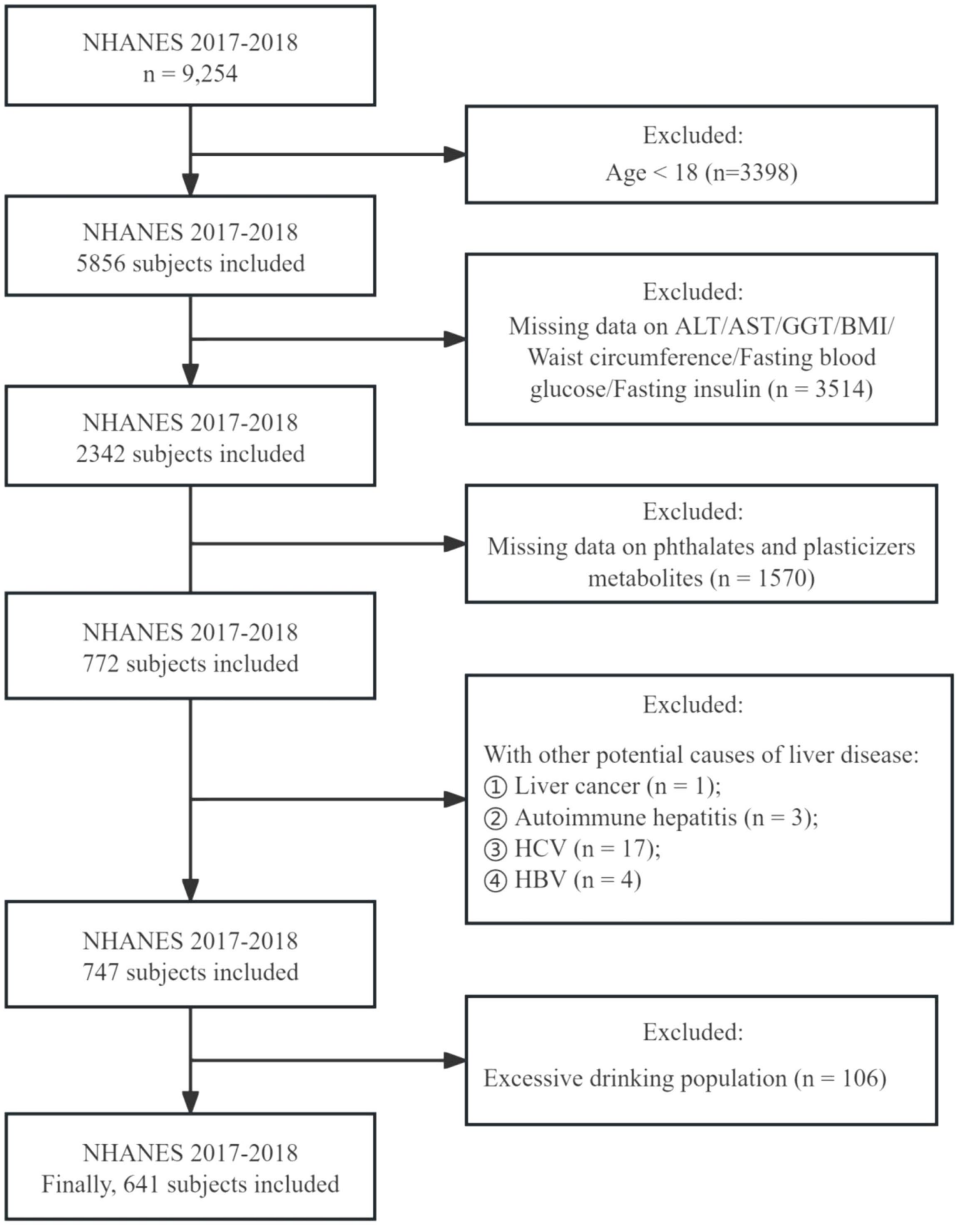
Flowchart of participants selection in the study.

In this study, we collected data from the NHANES database for the 2017 to 2018 cycles, involving 9,254 participants. Initially, as our focus was on adults, we excluded 3,398 individuals under the age of 18, leaving 5,856 adults. Of these, approximately two-fifths (2,342 participants) underwent testing for indicators used to assess the presence of NAFLD (ALT, AST, BMI, Diabetes, GGT, Waist circumference, Fasting insulin and Fasting glucose). However, only 772 participants were tested for the presence of phthalates and plasticizer metabolites in their urine. Additionally, we excluded patients with liver cancer, autoimmune hepatitis, and viral hepatitis (hepatitis C antibody, hepatitis C RNA positive; hepatitis B surface antigen positive). Furthermore, to eliminate the influence of excessive alcohol consumption on metabolic indicators and NAFLD diagnosis, we excluded 106 participants who were heavy drinkers. Excessive alcohol consumption was characterized by an average consumption exceeding 20 grams per day for men and 10 grams per day for women (25). Ultimately, 641 participants were enrolled (Figure 1).

### 2.2 Measurement of phthalates in urine

In our study, we evaluated ten major metabolites derived from seven different parent phthalates, including Mono-2-ethyl-5-carboxypentyl phthalate (MECPP), Mono-(2-ethyl-5-hydroxyhexyl) phthalate (MEHHP), Mono-(2-ethyl-5-oxohexyl) phthalate (MEOHP), Mono-(carboxyisononyl) phthalate (MCiNP), Mono-oxoisononyl phthalate (MOiNP), Mono-(carboxyisoctyl) phthalate (MCiOP), Mono-n-butyl phthalate (MnBP), Mono-isobutyl phthalate (MiBP), Mono-ethyl phthalate (MEP), Mono-(3-carboxypropyl) phthalate (MCPP). A thorough summary of each phthalate and its corresponding metabolites is provided in the Supplementary Figure S1. In this study, the urine samples were first processed by enzymolysis of glucosylated analytes, and then quantitatively detected by high performance liquid chromatography-electrospray ionization-tandem mass spectrometry (HPLC-ESI-MS/MS). In addition to this, the collection of these samples is often carried out in multiple locations across the country to ensure that the samples are representative.^2^

For sample analysis, values not detected due to being below the lower limit of detection (LOD) are populated with the LOD divided by the square root of 2. This approach aligns with the guidelines set by the National Center for Health Statistics (NCHS). A detailed analysis guide can be found at the following website: https://wwwn.cdc.gov/nchs/nhanes/analyticguidelines.aspx#analytic-guidelines.

### 2.3 Definition of NAFLD

Liver biopsy, traditionally the gold standard for diagnosing NAFLD, faces limitations due to its invasiveness and poor patient acceptance, making it less ideal for regular screening. To address this, researchers have introduced two effective models for disease screening: the HSI criteria and the US.FLI criteria. In the HSI prediction model, the AUC (95%CI) was 0.812 (0.801–0.824), and the cutoff values were 30 and 36. Values below 30, considering factors like ALT, AST, BMI, and gender, imply a minimal risk of NAFLD. In contrast, values above 36 suggest a high likelihood of the condition (26). In the US.FLI prediction model, the AUC (95%CI) was 0.80 (0.77–0.83), and the cutoff values were 30. If US.FLI ≥ 30, it was defined as NAFLD (27). HSI and US.FLI were calculated as follows:

HSI = 8*ALT (IU/L)/AST (IU/L) + body mass index (BMI, kg/m2) + 2 (if female) +2 (if type 2 diabetes).

HSI = 8*ALT (IU/L)/AST (IU/L) + body mass index (BMI, kg/m2) + 2 (if female) +2 (if type 2 diabetes).


(1) $$\mathrm{US.FLI}=\left(e^{\begin{array}{l} -0\mathrm{.8073*non-Hispanic}\;\mathrm{black+}\;0\mathrm{.3458*Mexican}\;\mathrm{Amerian}\;+\;\\ 0\mathrm{.0093*age}\;+\;0\mathrm{.6151*}\;\log_{e}\;\mathrm{(GGT)}\;+\;0\mathrm{.0249*waist}\;\mathrm{circumference}\;+\;\\ 1{\mathrm{.1792*log}}_{e}\mathrm{(insulin)}\;+\;0{\mathrm{.8242*log}}_{e}\mathrm{(glucose)}-14.7812 \end{array}}\right)\;/\;\left(1+e^{\begin{array}{l} -0\mathrm{.8073*non-Hispanic}\;\mathrm{black+}\;0\mathrm{.3458*Mexican}\;\mathrm{Amerian}\;+\;\\ 0\mathrm{.0093*age}\;+\;0\mathrm{.6151*}\;\log_{e}\;\mathrm{(GGT)}\;+\;0\mathrm{.0249*waist}\;\mathrm{circumference}\;+\\ 1{\mathrm{.1792*log}}_{e}\mathrm{(insulin)}\;+\;0{\mathrm{.8242*log}}_{e}\mathrm{(glucose)}-14.7812 \end{array}}\right)*100$$

VCTE is a specific liver elastography technique that assesses the stiffness of the liver by measuring its response to brief mechanical vibrations, defined by two variables, CAP and LSM. CAP was used to assess hepatic steatosis, and LSM was used to assess hepatic fibrosis. In this study, we referred to the conclusions of Eddowes et al., and defined NAFLD as LSM ≥ 8 kPa and CAP≥274 dB/m. In the VCTE criteria, the AUC (95%CI) was 0.87 (0.82–0.92) (28).

### 2.4 Definition of covariates

This study selected some of the demographic data, examination data, laboratory data, and variables defined by questionnaire data, including age (>18), gender (male/female), race (Mexican American/ Other Hispanic/ Non-Hispanic White/ Non-Hispanic Black/Non-Hispanic Asian/Other Race), education level (the highest degree obtained is either above or below high school) and other indicators. Smoking status was defined based on the total number of cigarettes smoked in a lifetime and whether they had smoked recently. Recreational physical activity was divided into vigorous activity, moderate activity, sedentary activity. Body mass index (BMI) was categorized into under/normal weight (<25 kg/m^2^), overweight (25–30 kg/m^2^), obese (> = 30 kg/m^2^). In addition, there was another indicator of obesity, waist circumference. Ensuring data accuracy, systolic blood pressure (SBP) measurements were conducted three times, with the average of these values being utilized for analysis. Diabetes was defined as a hemoglobin A1C (HbA1C) concentration > = 6.5% or fasting plasma glucose (FPG) > = 126 mg/dL or a person who had been told by a doctor that he or she had diabetes and was currently taking insulin. Other covariates, such as alanine aminotransferase (ALT), aspartate aminotransferase (AST), gamma-glutamyl transpeptidase (GGT), lactate dehydrogenase (LDH), albumin (ALB), globulin (GLO), were also included in this study. Covariates were selected based on their association with exposure and outcomes, informed by clinical experience and supported by the available literature.

### 2.5 Statistical analysis

The data in NHANES uses a complex multi-stage probabilistic sampling design. Therefore, this study referred to the methodology of the National Center for Health Statistics (NCHS) to accurately evaluate large population data in the United States, considering the primary sampling units (PSU) and the stratum variable (29). Due to the smallest subpopulation composition of phthalates and their metabolites, this study incorporated the use of subsample weights (wtsb2yr). All statistical analyses were performed using R (version 4.3.2), and Beckman Coulter DxAI platform v2.0.^3^ Furthermore, to address the skewed distribution, we transformed the urinary phthalate metabolites data using the natural logarithm (ln) method.

Continuous variables were described as median (IQR). Categorical variables were described as *n* (unweighted) (%). However, the calculation process is weighted analysis. Differences between groups were evaluated using Mann–Whitney U tests for continuous variables, depending on the skewness of the data. Chi-square tests were utilized for categorical variables. Multiple logistic regression analyses were conducted to explore the associations between Phthalates and suspected NAFLD, adjusting for potential confounders. All tests were two-tailed, and a *p*-value of less than 0.05 was considered statistically significant. Variables included in the logistic regression analysis underwent strict screening procedures. Specifically, due to the high correlation between the two variables (FPG vs. HbA1C; BMI vs. waist circumference, Pearson’s r ≥ 0.6) (Supplementary Figure S2), only one of the indicators was selected for adjustment, and variables with high correlation to the outcome variables were excluded (Supplementary Figure S3). Finally, we identify the variables that need to be adjusted for different models: model 1 was unadjusted; model 2, age and race were adjusted; model 3, age, race, recreational physical activity, SBP and HbA1C were adjusted.

## 3 Results

### 3.1 Baseline characteristics

Based on the inclusion and exclusion criteria previously mentioned, a total of 641 participants were enrolled in the study. These participants were evaluated using three different NAFLD prediction criteria: HSI > 36, US.FLI ≥ 30, and VCTE: LSMs >8 kPa and CAP ≥274 dB/m. Unfortunately, only 572 participants underwent VCTE assessment, owing to missing data on median CAP, median stiffness, or elastograph status. Table 1 presents the baseline characteristics. Under the HSI diagnostic criteria, participants with higher levels of obesity, fasting blood glucose, HbA1C, ALT, GGT, lower albumin, and higher globulin were more likely to have NAFLD. An increase in the concentration of phthalates and plasticizers metabolites in urine, specifically MEOHP, MEHHP, MECPP, MCiOP, and MCiNP, May be associated with NAFLD. Under the US.FLI diagnostic criteria, older age was more likely to be associated with the disease (42 vs. 53, *p* < 0.001). Similar to the HSI group, in addition to significant statistical differences in obesity, fasting blood glucose, HbA1C, ALT, GGT, albumin, and globulin, an increase in SBP and AST (117 vs. 125, *p* < 0.001; 18 vs. 20, *p* = 0.01) May also be related to NAFLD. Furthermore, higher concentrations of MnBP, MiBP, MCPP, and MEHHP might be linked to an increased likelihood of the disease. Under the VCTE criteria, trends were similar to those in the US.FLI results, but there were no statistical differences in AST, albumin, and globulin concentrations between the two groups. MEOHP, MEHHP, MCiOP, and MCiNP May be related to NAFLD. Additionally, Table 1 indicates that irrespective of the diagnostic criteria used, factors such as gender, education level, smoking status, and recreational physical activity showed no significant statistical differences, suggesting no association with NAFLD.

**Table 1:** Characteristics of NHANES participants by the NAFLD status, 2017–2018.

	Overall	HSI			US.FLI			VCTE		
		No	Yes		No	Yes		No	Yes	
**Characteristic**	*N*^1^ = 641 (100%)^2^	*N*^1^ = 228 (37%)^2^	*N^1^* = 413 (63%)^2^	*p-value* ^3^	*N*^1^ = 410 (65%)^2^	*N*^1^ = 231 (35%)^2^	*p-value^3^*	*N*^1^ = 317 (57%)^2^	*N*^1^ = 255 (43%)^2^	*p-value* ^3^
**Age**, years	46 (31, 61)	45 (28, 65)	46 (32, 61)	0.7	42 (28, 58)	53 (34, 64)	**<0.001**	41 (25, 58)	50 (37, 62)	**0.001**
**Gender**				0.4			0.9			0.7
Male	307 (50%)	120 (54%)	187 (48%)		194 (51%)	113 (49%)		150 (49%)	127 (53%)	
Female	334 (50%)	108 (46%)	226 (52%)		216 (49%)	118 (51%)		167 (51%)	128 (47%)	
**Race**				**0.048**			**<0.001**			**<0.001**
Mexican American	90 (8.8%)	18 (3.7%)	72 (12%)		36 (5.6%)	54 (15%)		27 (5.0%)	54 (14%)	
Other Hispanic	61 (6.6%)	18 (7.1%)	43 (6.2%)		41 (8.3%)	20 (3.3%)		35 (9.0%)	20 (3.0%)	
Non-Hispanic White	213 (61%)	77 (65%)	136 (59%)		129 (60%)	84 (63%)		95 (57%)	87 (65%)	
Non-Hispanic Black	155 (13%)	57 (13%)	98 (13%)		124 (16%)	31 (7.4%)		88 (15%)	54 (11%)	
Non-Hispanic Asian	92 (6.3%)	47 (8.4%)	45 (5.2%)		64 (7.2%)	28 (4.7%)		53 (7.2%)	33 (5.8%)	
Other Race	30 (3.9%)	11 (3.5%)	19 (4.2%)		16 (2.6%)	14 (6.4%)		19 (6.0%)	7 (1.7%)	
**Education level**				0.7			0.11			0.4
< High school	132 (12%)	43 (11%)	89 (12%)		74 (10%)	58 (14%)		60 (12%)	55 (10%)	
≥ High school	471 (88%)	165 (89%)	306 (88%)		305 (90%)	166 (86%)		228 (88%)	193 (90%)	
**Smoking status**				>0.9			0.2			0.3
Never	401 (63%)	147 (64%)	254 (63%)		275 (67%)	126 (55%)		219 (68%)	146 (59%)	
Former	139 (22%)	40 (22%)	99 (22%)		68 (18%)	71 (29%)		50 (18%)	70 (26%)	
Current	101 (15%)	41 (14%)	60 (15%)		67 (15%)	34 (16%)		48 (14%)	39 (15%)	
**Recreational physical activity**				0.4			0.063			0.2
Sedentary activity	85 (15%)	31 (11%)	54 (18%)		48 (12%)	37 (22%)		37 (11%)	37 (17%)	
Moderate activity	139 (37%)	56 (37%)	83 (36%)		92 (33%)	47 (45%)		65 (33%)	60 (41%)	
Vigorous activity	163 (49%)	66 (53%)	97 (46%)		126 (55%)	37 (34%)		101 (56%)	51 (42%)	
**BMI**, kg/m^2^	28 (24, 33)	23 (22, 25)	32 (29, 36)	**<0.001**	26 (23, 29)	34 (30, 39)	**<0.001**	25 (23, 29)	32 (28, 37)	**<0.001**
**BMI**				**<0.001**			**<0.001**			**<0.001**
Under/normal weight	169 (28%)	162 (71%)	7 (2.0%)		157 (39%)	12 (5.0%)		138 (46%)	21 (6.6%)	
Overweight	199 (33%)	66 (29%)	133 (35%)		149 (41%)	50 (17%)		101 (33%)	74 (31%)	
Obese	273 (40%)	0 (0%)	273 (63%)		104 (20%)	169 (78%)		78 (21%)	160 (62%)	
**Diabetes**				**0.002**			**<0.001**			**<0.001**
No	503 (86%)	186 (92%)	317 (83%)		369 (96%)	134 (68%)		274 (94%)	174 (77%)	
Yes	137 (14%)	41 (8.4%)	96 (17%)		40 (4.2%)	97 (32%)		42 (6.1%)	81 (23%)	
**Waist circumference, cm**	98 (87, 110)	85 (79, 91)	106 (97, 117)	**<0.001**	92 (82, 99)	113 (105, 123)	**<0.001**	89 (81, 99)	107 (98, 119)	**<0.001**
**SBP**, mmHg	121 (111, 129)	120 (109, 130)	122 (111, 129)	0.2	117 (108, 128)	125 (117, 133)	**<0.001**	115 (106, 127)	125 (116, 133)	**<0.001**
**HbA1C**, %	5.50 (5.20, 5.80)	5.40 (5.20, 5.60)	5.50 (5.20, 5.90)	**0.020**	5.40 (5.20, 5.60)	5.70 (5.40, 6.40)	**<0.001**	5.40 (5.20, 5.60)	5.60 (5.30, 6.10)	**<0.001**
**FPG**, mmol/L	103 (97, 111)	99 (94, 106)	106 (98, 113)	**<0.001**	99 (95, 107)	111 (104, 129)	**<0.001**	99 (95, 107)	108 (102, 118)	**<0.001**
**ALT**, U/L	18 (13, 25)	15 (11, 20)	20 (15, 29)	**<0.001**	16 (12, 22)	23 (17, 32)	**<0.001**	17 (13, 23)	21 (15, 30)	**0.010**
**AST**, U/L	19 (16, 23)	19 (16, 23)	19 (16, 23)	>0.9	18 (16, 22)	20 (18, 24)	**0.010**	19 (16, 23)	19 (16, 23)	0.9
**GGT**, U/L	19 (14, 28)	15 (13, 19)	23 (16, 30)	**<0.001**	17 (13, 21)	28 (22, 43)	**<0.001**	17 (13, 23)	24 (18, 33)	**<0.001**
**LDH**, IU/L	155 (136, 168)	154 (135, 171)	155 (137, 168)	>0.9	153 (133, 166)	157 (141, 172)	0.072	154 (135, 167)	155 (137, 169)	0.5
**ALB**, g/dL	4.10 (3.80, 4.30)	4.10 (3.90, 4.30)	4.00 (3.80, 4.20)	**0.005**	4.10 (3.90, 4.30)	4.00 (3.80, 4.20)	**0.031**	4.10 (3.90, 4.30)	4.10 (3.80, 4.20)	0.2
**GLO**, g/dL	3.00 (2.80, 3.30)	3.00 (2.70, 3.20)	3.08 (2.80, 3.40)	**0.019**	3.00 (2.70, 3.20)	3.10 (2.80, 3.50)	**0.039**	3.00 (2.70, 3.20)	3.10 (2.80, 3.40)	0.2
**AG_ratio**	1.34 (1.18, 1.52)	1.40 (1.26, 1.56)	1.30 (1.14, 1.48)	**0.004**	1.38 (1.22, 1.55)	1.28 (1.11, 1.46)	**0.030**	1.37 (1.23, 1.54)	1.31 (1.17, 1.50)	0.2
**Phthalates and plasticizers metabolites**, ng/mL										
**MEP**	28 (13, 65)	26 (11, 90)	30 (14, 59)	>0.9	26 (12, 76)	31 (17, 59)	0.6	28 (11, 74)	28 (14, 57)	0.7
**MnBP**	10 (5, 19)	8 (4, 17)	11 (5, 19)	0.3	9 (4, 18)	12 (6, 21)	**0.021**	10 (4, 20)	11 (6, 19)	0.6
**MiBP**	9 (4, 15)	7 (3, 12)	9 (5, 16)	0.2	8 (3, 12)	10 (5, 17)	**0.017**	9 (3, 16)	9 (5, 15)	0.7
**MCPP**	1.10 (0.60, 2.20)	0.99 (0.50, 2.30)	1.20 (0.70, 2.20)	0.3	1.00 (0.52, 2.20)	1.30 (0.80, 2.42)	**0.032**	1.02 (0.56, 2.10)	1.26 (0.70, 2.60)	0.069
**MOiNP**	1.40 (0.70, 2.50)	1.13 (0.50, 2.66)	1.40 (0.80, 2.40)	0.13	1.30 (0.60, 2.50)	1.50 (0.70, 2.30)	0.4	1.30 (0.60, 2.34)	1.50 (0.90, 2.50)	0.12
**MEOHP**	3.5 (1.6, 5.7)	2.5 (1.3, 5.2)	3.9 (1.9, 6.1)	**0.004**	3.1 (1.5, 6.1)	3.9 (2.1, 5.4)	0.071	2.8 (1.3, 5.6)	4.2 (2.4, 6.4)	**0.015**
**MEHHP**	5 (2, 9)	4 (2, 8)	6 (3, 9)	**0.008**	4 (2, 9)	6 (3, 9)	**0.034**	4 (2, 9)	6 (3, 10)	**0.021**
**MECPP**	9 (4, 14)	6 (3, 14)	10 (5, 14)	**0.029**	7 (4, 15)	10 (6, 14)	0.066	7 (3, 13)	10 (6, 15)	**0.027**
**MCiOP**	5 (3, 10)	5 (2, 9)	6 (3, 10)	**0.047**	5 (2, 9)	6 (3, 10)	0.2	5 (2, 10)	7 (3, 11)	**0.008**
**MCiNP**	1.40 (0.70, 2.40)	1.10 (0.50, 2.11)	1.50 (0.80, 2.60)	**0.018**	1.20 (0.61, 2.32)	1.50 (0.80, 2.97)	0.2	1.20 (0.60, 2.10)	1.75 (0.80, 3.00)	**0.031**
**Median Stiffness**	4.80 (4.00, 6.00)	4.40 (3.70, 5.59)	5.10 (4.10, 6.50)	**0.003**	4.40 (3.70, 5.50)	5.60 (4.61, 7.22)	**<0.001**	4.40 (3.80, 5.50)	5.40 (4.40, 6.80)	**<0.001**
**Median CAP**	261 (220, 307)	225 (192, 252)	286 (243, 330)	**<0.001**	239 (208, 276)	307 (268 346)	**<0.001**	225 (200, 244)	317 (291, 346)	**<0.001**

^1^*N* not Missing (unweighted). The calculation process is weighted analysis; ^2^Continuous variable: Median (IQR); Categorical variable: *n* (unweighted) (%). The calculation process is weighted analysis; ^3^Wilcoxon rank-sum test for complex survey samples; chi-squared test with Rao & Scott’s second-order correction; NAFLD, nonalcoholic fatty liver disease; HSI, hepatic steatosis index; US.FLI, the United States fatty liver index; VCTE, Vibration-controlled transient elastography. BMI, body mass index; SBP, systolic blood pressure; FPG, fasting plasma glucose; ALT, alanine aminotransferase; AST, aspartate aminotransferase; GGT, gamma-glutamyl transpeptidase; LDH, lactate dehydrogenase; ALB, albumin; GLO, globulin; AG_ratio is a calculated value: albumin (g/dL)/globulin (g/dL); MEP, Mono-ethyl phthalate; MnBP, Mono-n-butyl phthalate; MiBP, Mono-isobutyl phthalate; MCPP, Mono-(3-carboxypropyl) phthalate; MOiNP, Mono-oxoisononyl phthalate; MEOHP, Mono-(2-ethyl-5-oxohexyl) phthalate; MEHHP, Mono-(2-ethyl-5-hydroxyhexyl) phthalate; MECPP, Mono-2-ethyl-5-carboxypentyl phthalate; MCiOP, Mono (carboxyisooctyl) Phthalate; MCiNP, Mono-(carboxyisononyl) phthalate; CAP, controlled attenuated parameter. All of the statistically significant values (*p* < 0.05) have been highlighted in bold.

### 3.2 Association between baseline information and NAFLD

Univariate logistic regression analysis was conducted on variables exhibiting statistical differences in demographic, measurement, and laboratory test data as presented in Table 2. The objective was to identify factors potentially associated with outcomes in the same cohort of subjects when subjected to different diagnostic criteria for NAFLD. This analysis aids in the selection of variables for subsequent multivariate logistic regression.

**Table 2:** Univariate logistic regression analysis for all variables except urinary phthalates levels.

	HSI			US.FLI			VCTE		
Characteristic	OR^a^	95% CI^a^	*p-value*	OR^a^	95% CI^a^	*p-value*	OR^a^	95% CI^a^	*p-value*
**Age**, years	–	–	–	**1.02**	**1.01, 1.03**	**<0.001**	**1.02**	**1.01, 1.03**	**0.002**
**Race**									
Mexican American	Ref.	Ref.		Ref.	Ref.		Ref.	Ref.	
Other Hispanic	**0.27**	**0.08, 0.93**	**0.039**	**0.15**	**0.06, 0.35**	**<0.001**	**0.12**	**0.03, 0.46**	**0.006**
Non-Hispanic White	**0.29**	**0.11, 0.77**	**0.018**	**0.39**	**0.18, 0.81**	**0.017**	**0.41**	**0.17, 0.99**	**0.047**
Non-Hispanic Black	**0.33**	**0.13, 0.82**	**0.022**	**0.17**	**0.06, 0.47**	**0.003**	**0.25**	**0.10, 0.64**	**0.008**
Non-Hispanic Asian	**0.19**	**0.09, 0.43**	**0.001**	**0.24**	**0.11, 0.53**	**0.002**	**0.29**	**0.13, 0.64**	**0.006**
Other Race	0.38	0.10, 1.51	0.15	0.91	0.25, 3.30	0.9	**0.10**	**0.02, 0.41**	**0.005**
**BMI**, kg/m^2^	**2.77**	**2.18, 3.52**	**<0.001**	**1.29**	**1.22, 1.37**	**<0.001**	**1.20**	**1.13, 1.29**	**<0.001**
**Diabetes**									
No	Ref.	Ref.		Ref.	Ref.		Ref.	Ref.	
Yes	**2.23**	**1.40, 3.56**	**0.002**	**10.8**	**6.73, 17.5**	**<0.001**	**4.55**	**2.65, 7.81**	**<0.001**
**Waist circumference, cm**	**1.22**	**1.17, 1.27**	**<0.001**	**1.15**	**1.13, 1.18**	**<0.001**	**1.09**	**1.07, 1.12**	**<0.001**
**SBP**, mmHg	1.01	0.99, 1.02	0.4	**1.03**	**1.01, 1.04**	**<0.001**	**1.03**	**1.02, 1.05**	**<0.001**
**HbA1C**, %	**1.52**	**1.16, 1.99**	**0.005**	**3.02**	**2.19, 4.15**	**<0.001**	**2.12**	**1.39, 3.23**	**0.002**
**FPG**, mmol/L	**1.02**	**1.01, 1.03**	**<0.001**	**1.06**	**1.04, 1.08**	**<0.001**	**1.03**	**1.01, 1.04**	**0.004**
**ALT**, U/L	**1.07**	**1.03, 1.12**	**0.002**	**1.09**	**1.05, 1.12**	**<0.001**	**1.03**	**1.00, 1.06**	**0.027**
**AST**, U/L	–	–	–	1.03	1.0, 1.06	0.092	–	–	–
**GGT**, U/L	1.02	0.98, 1.06	0.2	**1.06**	**1.01, 1.12**	**0.034**	**1.02**	**1.00, 1.04**	**0.026**
**ALB**, g/dL	**0.28**	**0.13, 0.60**	**0.003**	**0.38**	**0.17, 0.86**	**0.023**	–	–	–
**GLO**, g/dL	**2.31**	**1.16, 4.58**	**0.021**	**2.40**	**1.05, 5.46**	**0.039**	–	–	–
**AG_ratio**	**0.17**	**0.06, 0.50**	**0.003**	**0.19**	**0.04, 0.84**	**0.031**	–	–	–

a OR = Odds Ratio, CI = Confidence Interval; NAFLD, nonalcoholic fatty liver disease; HSI, hepatic steatosis index; US.FLI, the United States fatty liver index; VCTE, Vibration-controlled transient elastography. BMI, body mass index; SBP, systolic blood pressure; FPG, fasting plasma glucose; ALT, alanine aminotransferase; AST, aspartate aminotransferase; LDH, lactate dehydrogenase; ALB, albumin; GLO, globulin; AG_ratio is a calculated value: albumin (g/dL)/globulin (g/dL). All of the statistically significant values (*p* < 0.05) have been highlighted in bold.

As demonstrated in Table 2, heterogeneous results were obtained when the same cohort underwent diagnosis using varying predictive criteria. Under the HSI criteria, physical measurements and laboratory indicators such as BMI, waist circumference, diabetes status, HbA1C, fasting plasma glucose, ALT, and globulin levels were positively correlated with NAFLD, all showing statistical significance. In contrast, serum albumin levels were inversely related to the disease (OR = 0.28, 95%CI = 0.13–0.60, *p* = 0.003). Additionally, compared to Mexican Americans, other races showed a negative correlation with the disease. Under the US.FLI criteria, the older the age, the greater the positive association with the disease (OR = 1.02, 95%CI = 1.01–1.03, *p* < 0.001). Furthermore, GGT was positively correlated with NAFLD (OR = 1.06, 95% CI = 1.01–1.12, *p* = 0.034), with other results similar to those under the HSI criteria. Under the VCTE criteria, age, race, BMI, diabetes status, waist circumference, systolic blood pressure, fasting plasma glucose, HbA1C, ALT, and GGT were all positively correlated with NAFLD, as detailed in Table 2.

### 3.3 Association between urinary phthalates and NAFLD

The final metabolites of various phthalates May have different associations with NAFLD under different diagnostic criteria (Table 3). We observed that under the HSI criteria, there were significant positive correlations between NAFLD and the MEOHP, MEHHP, MECPP, and MCiOP (OR = 1.46, 95% CI = 1.11–1.93, *p* = 0.011; OR = 1.46, 95% CI = 1.10–1.95, *p* = 0.013; OR = 1.44, 95% CI = 1.04–2.00, *p* = 0.030; OR = 1.26, 95% CI = 1.01–1.58, *p* = 0.043). Under the US.FLI criteria, in addition to MEOHP, MEHHP, and MECPP, MnBP and MiBP also showed a positive correlation with NAFLD (OR = 1.29, 95% CI = 1.02–1.61, *p* = 0.033; OR = 1.26, 95% CI = 1.03–1.54, *p* = 0.026), while the correlation with MCiOP was not observed. Similarly to the HSI criteria, under the VCTE standards, MEOHP, MEHHP, MECPP, and MCiOP were positively correlated with the disease (OR = 1.47, 95% CI = 1.11–1.94, *p* = 0.010; OR = 1.45, 95% CI = 1.07–1.96, *p* = 0.020; OR = 1.53, 95% CI = 1.10–2.13, *p* = 0.014). Additionally, MCiNP also displayed a statistically significant positive correlation with the disease (OR = 1.36, 95% CI = 1.03–1.79, *p* = 0.035).

**Table 3:** Univariate logistic regression analysis for NAFLD according to urinary phthalates levels.

	HSI			US.FLI			VCTE		
Characteristic	OR^a^	95% CI^a^	*p-value*	OR^a^	95% CI^a^	*p-value*	OR^a^	95% CI^a^	*p-value*
MEP	0.97	0.81, 1.17	0.8	1.02	0.85, 1.24	0.8	0.93	0.79, 1.09	0.3
MnBP	1.20	0.92, 1.58	0.2	**1.29**	**1.02, 1.61**	**0.033**	1.18	0.84, 1.66	0.3
MiBP	1.12	0.88, 1.41	0.3	**1.26**	**1.03, 1.54**	**0.026**	1.06	0.83, 1.34	0.6
MCPP	1.12	0.82, 1.55	0.4	1.31	1.00, 1.71	0.053	1.30	1.0, 1.70	0.054
MOiNP	1.26	0.96, 1.66	0.090	1.13	0.87, 1.49	0.3	1.31	0.99, 1.73	0.056
MEOHP	**1.46**	**1.11, 1.93**	**0.011**	**1.29**	**1.03, 1.60**	**0.028**	**1.47**	**1.11, 1.94**	**0.010**
MEHHP	**1.46**	**1.10, 1.95**	**0.013**	**1.32**	**1.05, 1.67**	**0.021**	**1.45**	**1.07, 1.96**	**0.020**
MECPP	**1.44**	**1.04, 2.00**	**0.030**	**1.34**	**1.05, 1.72**	**0.021**	**1.53**	**1.10, 2.13**	**0.014**
MCiOP	**1.26**	**1.01, 1.58**	**0.043**	1.17	0.92, 1.48	0.2	**1.40**	**1.11, 1.77**	**0.008**
MCiNP	1.17	0.96, 1.43	0.11	1.11	0.82, 1.49	0.5	**1.36**	**1.03, 1.79**	**0.035**

a OR = Odds Ratio, CI = Confidence Interval; NAFLD, nonalcoholic fatty liver disease; HSI, hepatic steatosis index; US.FLI, the United States fatty liver index; VCTE, Vibration-controlled transient elastography. MEP, Mono-ethyl phthalate; MnBP, Mono-n-butyl phthalate; MiBP, Mono-isobutyl phthalate; MCPP, Mono-(3-carboxypropyl) phthalate; MOiNP, Mono-oxoisononyl phthalate; MEOHP, Mono-(2-ethyl-5-oxohexyl) phthalate; MEHHP, Mono-(2-ethyl-5-hydroxyhexyl) phthalate; MECPP, Mono-2-ethyl-5-carboxypentyl phthalate; MCiOP, Mono (carboxyisooctyl) Phthalate; MCiNP, Mono-(carboxyisononyl) phthalate. All of the statistically significant values (*p* < 0.05) have been highlighted in bold.

The above results illustrate the independent association of each indicator with NAFLD under different disease prediction criteria. However, it is yet to be ascertained whether these indicators are interrelated, and consequently, if they collectively contribute to the pathogenesis of NAFLD. Furthermore, to ascertain if the effect exhibited concentration-dependence, we classified phthalates into four distinct concentration gradients. Consequently, we conducted a multivariate logistic regression analysis to account for the combined impact of various factors on the outcome.

### 3.4 Correlation between phthalates and NAFLD based on logistic regression model

Three logistic regression models were constructed: model 1, no covariates were adjusted (Table 4); model 2, age and race were adjusted (Table 5); model 3, age, race, recreational physical activity, systolic blood pressure and HbA1C were adjusted (Table 6). We investigated the relationship between varying concentration gradients of urinary phthalates and NAFLD in a cohort, using different diagnostic criteria. Under the HSI criteria, elevated concentrations of MEP, MCPP, MOiNP, MEOHP, MEHHP, MECPP, MCiOP, and MCiNP exhibited positive correlations with NAFLD, suggesting an increased disease likelihood at higher levels. Specifically, most of this correlation is concentrated in the third quartile (Q3) versus the first (Q1), regardless of model 1 (M1) or model 2 (M2). Odds Ratios for Q3 vs. Q1 were as follows: MEP (M1: 1.93; M2: 1.89), MCPP (M1: 2.27; M2: 2.38), MOiNP (M1: 2.86; M2: 2.93), MEOHP (M1: 2.68; M2: 2.81), MEHHP (M1: 2.75; M2: 2.85), MECPP (M1: 4.19; M2: 4.22), with *p*-values <0.05. MCiOP and MCiNP also showed significant positive correlation, particularly in Q2 vs. Q1 (Tables 4, 5). Under the US.FLI standard, similar trends were observed. MEP, MnBP, MiBP, MCPP, MEOHP, MEHHP, and MECPP displayed positive correlations with NAFLD in model 1 and model 2. The higher the urinary phthalates, the higher the positive correlation with NALFD. Furthermore, MiBP and MCPP demonstrated significant associations across multiple quartiles, indicating a consistent trend. The VCTE criteria further corroborated these findings. Compounds like MEOHP, MEHHP, MECPP, and MCiOP exhibited positive correlations with NAFLD (Tables 4, 5).

**Table 4:** Model 1—univariate logistic regression analysis for NAFLD according to urinary phthalates quantile levels.

	HSI			US.FLI			VCTE		
Characteristic	OR^a^	95% CI^a^	*p-value*	OR^a^	95% CI^a^	*p-value*	OR^a^	95% CI^a^	*p-value*
**MEP**									
Q1	Ref.	Ref.		Ref.	Ref.		Ref.	Ref.	
Q2	1.44	0.78, 2.67	0.2	1.48	0.76, 2.87	0.2	1.63	0.76, 3.47	0.2
Q3	**1.93**	**1.23, 3.01**	**0.008**	**2.30**	**1.21, 4.35**	**0.015**	1.64	0.80, 3.37	0.2
Q4	1.01	0.47, 2.20	>0.9	1.06	0.40, 2.79	0.9	1.03	0.48, 2.20	>0.9
**MnBP**									
Q1	Ref.	Ref.		Ref.	Ref.		Ref.	Ref.	
Q2	0.92	0.50, 1.68	0.8	1.75	0.85, 3.58	0.12	1.57	0.78, 3.19	0.2
Q3	1.27	0.57, 2.82	0.5	**2.17**	**1.07, 4.39**	**0.034**	1.55	0.62, 3.85	0.3
Q4	1.16	0.48, 2.79	0.7	1.90	0.88, 4.12	0.10	1.21	0.39, 3.73	0.7
**MiBP**									
Q1	Ref.	Ref.		Ref.	Ref.		Ref.	Ref.	
Q2	1.20	0.87, 1.67	0.2	1.80	0.91, 3.55	0.085	1.22	0.74, 2.01	0.4
Q3	1.64	0.64, 4.17	0.3	1.77	0.82, 3.83	0.13	1.46	0.60, 3.59	0.4
Q4	1.71	0.87, 3.37	0.11	**2.36**	**1.11, 5.00**	**0.029**	1.05	0.46, 2.39	>0.9
**MCPP**									
Q1	Ref.	Ref.		Ref.	Ref.		Ref.	Ref.	
Q2	1.54	0.86, 2.77	0.13	**2.20**	**1.25, 3.89**	**0.010**	1.47	0.82, 2.64	0.2
Q3	**2.27**	**1.22, 4.24**	**0.014**	2.01	0.92, 4.37	0.075	1.55	0.96, 2.49	0.069
Q4	1.29	0.58, 2.86	0.5	**2.26**	**1.06, 4.80**	**0.036**	1.80	0.86, 3.79	0.11
**MOiNP**									
Q1	Ref.	Ref.		Ref.	Ref.		Ref.	Ref.	
Q2	**2.34**	**1.07, 5.09**	**0.035**	1.24	0.66, 2.33	0.5	1.59	0.74, 3.42	0.2
Q3	**2.86**	**1.54, 5.31**	**0.003**	1.86	0.91, 3.84	0.085	1.88	0.60, 5.85	0.3
Q4	1.38	0.59, 3.19	0.4	1.07	0.43, 2.64	0.9	1.46	0.64, 3.36	0.3
**MEOHP**									
Q1	Ref.	Ref.		Ref.	Ref.		Ref.	Ref.	
Q2	1.06	0.55, 2.06	0.8	1.62	0.79, 3.34	0.2	1.22	0.62, 2.41	0.5
Q3	**2.68**	**1.59, 4.53**	**0.001**	**3.01**	**1.43, 6.33**	**0.007**	**3.04**	**1.40, 6.60**	**0.009**
Q4	**1.79**	**1.08, 2.95**	**0.027**	1.30	0.66, 2.54	0.4	2.01	0.91, 4.41	0.077
**MEHHP**									
Q1	Ref.	Ref.		Ref.	Ref.		Ref.	Ref.	
Q2	1.13	0.59, 2.17	0.7	1.53	0.80, 2.92	0.2	1.08	0.57, 2.05	0.8
Q3	**2.75**	**1.49, 5.08**	**0.004**	**2.88**	**1.40, 5.92**	**0.008**	**3.25**	**1.55, 6.83**	**0.005**
Q4	**1.98**	**1.02, 3.85**	**0.044**	1.76	0.90, 3.45	0.092	2.04	0.79, 5.21	0.13
**MECPP**									
Q1	Ref.	Ref.		Ref.	Ref.		Ref.	Ref.	
Q2	1.52	0.78, 2.96	0.2	1.80	0.81, 4.00	0.14	2.03	0.99, 4.18	0.054
Q3	**4.19**	**2.42, 7.23**	**<0.001**	**3.58**	**1.77, 7.24**	**0.002**	**3.37**	**1.31, 8.64**	**0.016**
Q4	1.61	0.79, 3.29	0.2	1.40	0.67, 2.92	0.3	2.33	0.90, 6.02	0.076
**MCiOP**									
Q1	Ref.	Ref.		Ref.	Ref.		Ref.	Ref.	
Q2	**2.24**	**1.28, 3.91**	**0.008**	1.44	0.75, 2.78	0.2	**2.01**	**1.07, 3.78**	**0.032**
Q3	1.99	0.91, 4.36	0.079	1.74	0.86, 3.54	0.11	**3.36**	**1.64, 6.88**	**0.003**
Q4	1.91	0.95, 3.85	0.068	1.51	0.60, 3.81	0.3	**2.30**	**1.02, 5.19**	**0.045**
**MCiNP**									
Q1	Ref.	Ref.		Ref.	Ref.		Ref.	Ref.	
Q2	**2.24**	**1.19, 4.22**	**0.017**	1.59	0.75, 3.39	0.2	1.50	0.79, 2.87	0.2
Q3	1.45	0.61, 3.46	0.4	1.10	0.37, 3.31	0.9	1.66	0.74, 3.74	0.2
Q4	**2.21**	**1.21, 4.04**	**0.014**	2.05	0.76, 5.52	0.14	**2.58**	**1.25, 5.34**	**0.015**

a OR = Odds Ratio, CI = Confidence Interval; NAFLD, nonalcoholic fatty liver disease; HSI, hepatic steatosis index; US.FLI, the United States fatty liver index; VCTE, Vibration-controlled transient elastography. MEP, Mono-ethyl phthalate; MnBP, Mono-n-butyl phthalate; MiBP, Mono-isobutyl phthalate; MCPP, Mono-(3-carboxypropyl) phthalate; MOiNP, Mono-oxoisononyl phthalate; MEOHP, Mono-(2-ethyl-5-oxohexyl) phthalate; MEHHP, Mono-(2-ethyl-5-hydroxyhexyl) phthalate; MECPP, Mono-2-ethyl-5-carboxypentyl phthalate; MCiOP, Mono (carboxyisooctyl) Phthalate; MCiNP, Mono-(carboxyisononyl) phthalate. All of the statistically significant values (*p* < 0.05) have been highlighted in bold.

**Table 5:** Model 2—multivariate logistic regression analysis for NAFLD according to urinary phthalates levels.

	HSI			US.FLI			VCTE		
Characteristic	OR^a^	95% CI^a^	*p-value*	OR^a^	95% CI^a^	*p-value*	OR^a^	95% CI^a^	*p-value*
**MEP**									
Q1	Ref.	Ref.		Ref.	Ref.		Ref.	Ref.	
Q2	1.42	0.72, 2.78	0.3	1.72	0.78, 3.82	0.15	1.86	0.72, 4.81	0.2
Q3	**1.89**	**1.11, 3.20**	**0.025**	**2.85**	**1.43, 5.71**	**0.010**	1.97	0.84, 4.63	0.10
Q4	0.98	0.38, 2.54	>0.9	1.39	0.45, 4.28	0.5	1.17	0.44, 3.12	0.7
**MnBP**									
Q1	Ref.	Ref.		Ref.	Ref.		Ref.	Ref.	
Q2	0.89	0.46, 1.74	0.7	1.70	0.71, 4.07	0.2	1.56	0.62, 3.93	0.3
Q3	1.25	0.51, 3.09	0.6	2.34	1.00, 5.48	0.051	1.67	0.49, 5.61	0.3
Q4	1.20	0.47, 3.07	0.7	**2.50**	**1.03, 6.10**	**0.045**	1.35	0.32, 5.66	0.6
**MiBP**									
Q1	Ref.	Ref.		Ref.	Ref.		Ref.	Ref.	
Q2	1.25	0.91, 1.72	0.15	**2.45**	**1.14, 5.27**	**0.028**	1.45	0.76, 2.79	0.2
Q3	1.70	0.62, 4.64	0.3	**2.62**	**1.00, 6.85**	**0.050**	1.80	0.56, 5.82	0.3
Q4	1.70	0.82, 3.49	0.13	**3.73**	**1.62, 8.57**	**0.008**	1.40	0.48, 4.04	0.5
**MCPP**									
Q1	Ref.	Ref.		Ref.	Ref.		Ref.	Ref.	
Q2	1.64	0.84, 3.20	0.12	**2.68**	**1.15, 6.26**	**0.029**	1.67	0.76, 3.64	0.2
Q3	**2.38**	**1.19, 4.77**	**0.021**	2.31	0.84, 6.39	0.091	1.57	0.80, 3.11	0.2
Q4	1.44	0.60, 3.44	0.4	**3.03**	**1.23, 7.46**	**0.024**	1.97	0.76, 5.07	0.13
**MOiNP**									
Q1	Ref.	Ref.		Ref.	Ref.		Ref.	Ref.	
Q2	2.27	0.94, 5.48	0.063	1.27	0.57, 2.87	0.5	1.77	0.63, 4.96	0.2
Q3	**2.93**	**1.52, 5.62**	**0.006**	2.17	0.84, 5.60	0.093	2.23	0.50, 9.87	0.2
Q4	1.40	0.56, 3.50	0.4	1.31	0.47, 3.69	0.5	1.80	0.64, 5.10	0.2
**MEOHP**									
Q1	Ref.	Ref.		Ref.	Ref.		Ref.	Ref.	
Q2	1.04	0.52, 2.08	0.9	1.72	0.69, 4.32	0.2	1.31	0.51, 3.35	0.5
Q3	**2.81**	**1.62, 4.88**	**0.003**	**3.31**	**1.27, 8.64**	**0.023**	**3.26**	**1.19, 8.94**	**0.029**
Q4	1.78	0.98, 3.22	0.055	1.61	0.73, 3.57	0.2	2.39	0.83, 6.84	0.090
**MEHHP**									
Q1	Ref.	Ref.		Ref.	Ref.		Ref.	Ref.	
Q2	1.13	0.55, 2.29	0.7	1.61	0.67, 3.86	0.2	1.15	0.48, 2.78	0.7
Q3	**2.85**	**1.47, 5.52**	**0.007**	**3.47**	**1.28, 9.39**	**0.022**	**3.98**	**1.43, 11.1**	**0.016**
Q4	2.05	0.95, 4.43	0.064	**2.25**	**1.03, 4.92**	**0.044**	2.48	0.70, 8.79	0.13
**MECPP**									
Q1	Ref.	Ref.		Ref.	Ref.		Ref.	Ref.	
Q2	1.45	0.71, 2.97	0.3	1.67	0.60, 4.66	0.3	2.09	0.88, 4.95	0.081
Q3	**4.22**	**2.44, 7.29**	**<0.001**	**3.96**	**1.64, 9.55**	**0.009**	**3.52**	**1.01, 12.2**	**0.049**
Q4	1.60	0.71, 3.57	0.2	1.66	0.64, 4.31	0.2	2.70	0.80, 9.05	0.092
**MCiOP**									
Q1	Ref.	Ref.		Ref.	Ref.		Ref.	Ref.	
Q2	**2.20**	**1.22, 3.96**	**0.016**	1.60	0.68, 3.80	0.2	**2.45**	**1.03, 5.83**	**0.045**
Q3	2.06	0.88, 4.87	0.086	2.05	0.82, 5.17	0.11	**4.55**	**1.93, 10.7**	**0.005**
Q4	1.90	0.88, 4.07	0.088	1.70	0.61, 4.73	0.2	2.66	0.88, 7.99	0.073
**MCiNP**									
Q1	Ref.	Ref.		Ref.	Ref.		Ref.	Ref.	
Q2	**2.24**	**1.11, 4.49**	**0.030**	1.57	0.71, 3.47	0.2	1.29	0.66, 2.54	0.4
Q3	1.53	0.60, 3.88	0.3	1.29	0.36, 4.59	0.6	1.73	0.58, 5.14	0.3
Q4	**2.36**	**1.22, 4.58**	**0.018**	2.53	0.85, 7.51	0.083	2.79	0.94, 8.32	0.061

a OR = Odds Ratio, CI = Confidence Interval; NAFLD, nonalcoholic fatty liver disease; HSI, hepatic steatosis index; US.FLI, the United States fatty liver index; VCTE, Vibration-controlled transient elastography. MEP, Mono-ethyl phthalate; MnBP, Mono-n-butyl phthalate; MiBP, Mono-isobutyl phthalate; MCPP, Mono-(3-carboxypropyl) phthalate; MOiNP, Mono-oxoisononyl phthalate; MEOHP, Mono-(2-ethyl-5-oxohexyl) phthalate; MEHHP, Mono-(2-ethyl-5-hydroxyhexyl) phthalate; MECPP, Mono-2-ethyl-5-carboxypentyl phthalate; MCiOP, Mono (carboxyisooctyl) Phthalate; MCiNP, Mono-(carboxyisononyl) phthalate. All of the statistically significant values (*p* < 0.05) have been highlighted in bold.

**Table 6:** Model 3—multivariate logistic regression analysis for NAFLD according to urinary phthalates levels.

	HSI			US.FLI			VCTE		
Characteristic	OR^a^	95% CI^a^	*p-value*	OR^a^	95% CI^a^	*p-value*	OR^a^	95% CI^a^	*p-value*
**MEP**									
Q1	Ref.	Ref.		Ref.	Ref.		Ref.	Ref.	
Q2	2.28	0.29, 18.3	0.2	3.66	0.16, 86.1	0.2	4.43	0.27, 73.4	0.2
Q3	1.99	0.51, 7.67	0.2	2.99	0.13, 71.1	0.3	2.86	0.20, 41.3	0.2
Q4	0.93	0.12, 7.10	0.9	2.45	0.10, 57.2	0.3	2.43	0.09, 63.8	0.4
**MnBP**									
Q1	Ref.	Ref.		Ref.	Ref.		Ref.	Ref.	
Q2	0.73	0.16, 3.23	0.5	1.40	0.10, 19.8	0.6	1.69	0.17, 16.8	0.4
Q3	2.27	0.23, 22.4	0.3	4.71	0.22, 99.6	0.2	3.73	0.32, 43.5	0.15
Q4	1.18	0.16, 8.92	0.8	2.54	0.24, 26.4	0.2	2.05	0.10, 40.8	0.4
**MiBP**									
Q1	Ref.	Ref.		Ref.	Ref.		Ref.	Ref.	
Q2	1.29	0.40, 4.14	0.4	1.77	0.28, 11.2	0.3	1.50	0.30, 7.40	0.4
Q3	2.76	0.36, 21.2	0.2	3.88	0.53, 28.6	0.10	3.12	0.19, 50.1	0.2
Q4	1.21	0.09, 15.6	0.8	3.88	0.24, 61.6	0.2	2.02	0.46, 8.88	0.2
**MCPP**									
Q1	Ref.	Ref.		Ref.	Ref.		Ref.	Ref.	
Q2	1.10	0.19, 6.55	0.8	1.38	0.25, 7.48	0.5	1.74	0.36, 8.41	0.3
Q3	2.58	0.30, 22.4	0.2	3.17	0.17, 57.8	0.2	2.48	0.59, 10.5	0.11
Q4	1.71	0.21, 14.2	0.4	2.36	0.19, 29.7	0.3	2.84	0.27, 30.3	0.2
**MOiNP**									
Q1	Ref.	Ref.		Ref.	Ref.		Ref.	Ref.	
Q2	3.26	0.22, 49.2	0.2	1.07	0.12, 9.71	>0.9	1.67	0.19, 15.0	0.4
Q3	2.66	0.30, 23.2	0.2	1.57	0.17, 14.8	0.5	1.87	0.14, 25.5	0.4
Q4	1.59	0.15, 17.4	0.5	1.01	0.09, 11.6	>0.9	1.62	0.25, 10.3	0.4
**MEOHP**									
Q1	Ref.	Ref.		Ref.	Ref.		Ref.	Ref.	
Q2	0.91	0.10, 7.95	0.9	1.73	0.26, 11.5	0.3	1.28	0.07, 22.3	0.7
Q3	2.40	0.35, 16.6	0.2	2.32	0.10, 54.9	0.4	3.77	0.41, 34.7	0.12
Q4	2.35	0.81, 6.88	0.075	2.65	0.15, 47.9	0.3	4.60	0.41, 51.5	0.11
**MEHHP**									
Q1	Ref.	Ref.		Ref.	Ref.		Ref.	Ref.	
Q2	0.99	0.14, 7.13	>0.9	1.58	0.21, 11.9	0.4	0.86	0.08, 9.89	0.8
Q3	2.67	0.47, 15.1	0.13	2.15	0.14, 33.6	0.4	4.84	0.50, 46.9	0.10
Q4	2.99	0.69, 13.0	0.085	3.55	0.27, 46.1	0.2	4.78	0.39, 58.7	0.12
**MECPP**									
Q1	Ref.	Ref.		Ref.	Ref.		Ref.	Ref.	
Q2	0.81	0.11, 5.87	0.7	0.88	0.08, 9.39	0.8	1.36	0.18, 10.3	0.6
Q3	5.25	0.82, 33.7	0.062	4.31	0.46, 40.0	0.11	4.50	0.34, 59.2	0.13
Q4	1.50	0.17, 12.8	0.5	1.65	0.20, 13.8	0.4	4.21	0.44, 40.5	0.11
**MCiOP**									
Q1	Ref.	Ref.		Ref.	Ref.		Ref.	Ref.	
Q2	2.12	0.35, 12.9	0.2	1.24	0.19, 8.03	0.7	2.05	0.31, 13.5	0.2
Q3	1.69	0.25, 11.4	0.4	1.83	0.27, 12.5	0.3	**4.22**	**1.10, 16.2**	**0.044**
Q4	1.99	0.20, 20.1	0.3	2.25	0.24, 21.2	0.3	3.04	0.49, 18.8	0.12
**MCiNP**									
Q1	Ref.	Ref.		Ref.	Ref.		Ref.	Ref.	
Q2	2.29	0.32, 16.6	0.2	1.38	0.08, 24.2	0.7	0.94	0.22, 3.93	0.9
Q3	2.65	0.35, 19.9	0.2	3.20	0.16, 63.3	0.2	2.73	0.39, 19.1	0.2
Q4	2.22	0.70, 7.00	0.10	3.77	0.26, 54.3	0.2	3.30	0.19, 57.0	0.2

a OR = Odds Ratio, CI = Confidence Interval; MEP, Mono-ethyl phthalate; MnBP, Mono-n-butyl phthalate; MiBP, Mono-isobutyl phthalate; MCPP, Mono-(3-carboxypropyl) phthalate; MOiNP, Mono-oxoisononyl phthalate; MEOHP, Mono-(2-ethyl-5-oxohexyl) phthalate; MEHHP, Mono-(2-ethyl-5-hydroxyhexyl) phthalate; MECPP, Mono-2-ethyl-5-carboxypentyl phthalate; MCiOP, Mono (carboxyisooctyl) Phthalate; MCiNP, Mono-(carboxyisononyl) phthalate. All of the statistically significant values (*p* < 0.05) have been highlighted in bold.

However, when all covariates were adjusted for multivariate logistic regression analysis, we found that no matter which prediction method was used for diagnosis (HSI or US.FLI) in our cohort, all the phthalates mentioned above lost their relevance. Only under the criterion of VCTE, a higher concentration of MCiOP in urine was positively correlated with NAFLD (OR = 4.22, 95% CI = 1.10–16.2, *p* = 0.044), and this correlation was found in models 1,2, and 3 (Table 6). If participants used VCTE to predict disease outcomes, those with more MCiOP in their urine were more likely to develop the disease, suggesting that MCiOP May be a risk factor for NAFLD, although this was cross-sectional study.

## 4 Discussion

This study, utilizing NHANES database data, reveals that higher concentrations of specific phthalate metabolites correlate positively with NAFLD, with the correlation’s strength varying by diagnostic criteria. Notably, only under VCTE criteria did the Mono-(carboxyisoctyl) phthalate (MCiOP) correlate positively with NAFLD. Based on what we know, this research innovatively compares multiple NAFLD diagnostic methods (HSI>36, US.FLI ≥ 30 and VCTE:LSM ≥ 8 kPa or CAP≥274 dB/m), highlighting the impact of diagnostic method selection on correlations. Furthermore, our analysis extends beyond traditional phthalates and their metabolites, incorporating a broader range of substitutes to provide a more comprehensive understanding of how various metabolites influence disease.

Phthalates May affect NAFLD through multiple pathways. Cell experiments show that phthalates exposure can trigger lipid accumulation and amplify oxidative stress in hepatocytes via the suppression of the JAK2/STAT5 pathway in BRL-3A cells, raising the risk of NAFLD (30). In animal studies, phthalates have been shown to facilitate macrophage polarization by simultaneously activating PPARα and PPARγ. This coordinated activation contributes to the disruption of lipid homeostasis within the liver (31). Moreover, phthalates can also cause liver cell damage by affecting oxidative stress metabolism (32). In population-based studies, researchers utilizing data from The Korean National Environmental Health Survey (KoNEHS) indicated that the third and fourth quartiles of MEHHP were positively correlated with the prevalence of NAFLD among Korean adults (OR = 1.33, 95% CI = 1.00–1.78; OR = 1.39, 95% CI = 1.00–1.92), employing the HSI diagnostic criteria (18). Another analysis, using data from the NHANES 2003–2016 database of a representative US population, based on the HSI diagnostic criteria, showing that MEOHP (OR = 1.56, 95% CI = 1.08–2.24), MEHHP (OR = 1.55, 95% CI = 1.09–2.21), and MECPP (OR = 1.44, 95% CI = 1.06–1.95) are associated with NAFLD. However, only MEHHP (OR = 1.98, 95% CI = 1.32–2.97) was found to be related to NAFLD under the US.FLI diagnostic method (19). Recently, another study included individuals from the NHANES 2017 to 2018 cycles and discovered a significant correlation between MECPP (OR = 2.719, 95% CI = 1.296–5.700) and MEHHP (OR = 2.073, 95% CI = 1.111–3.867) with NAFLD under the VCTE criteria. Nonetheless, this correlation vanished under HSI and US.FLI standards. Similarly, He et al. found that patients with higher urinary DEHP metabolite concentrations had an increased risk of NAFLD in the partially adjusted logistic regression model (OR = 1.22, 95%CI = 1.09–1.36), but lost the association in the fully adjusted model based on the VCTE (20, 21). These contradictory findings indicate that the relationship between phthalate metabolites and NAFLD is influenced by two factors: the selection of the population and the choice of diagnostic methods. In our research, fully adjusted multivariate logistic regression results demonstrated that three metabolites of DEHP (MECPP, MEHHP, and MEOHP) were not associated with NAFLD, similar to the above conclusions. Due to legislative impacts, exposure to DEHP has been decreasing over the years, with its alternatives, such as DINP—a less toxic substitute—increasingly being used (33). Utilizing three predictive methods, HSI, US.FLI, and VCTE, our analysis of various phthalates and their substitutes’ metabolites found that only when the VCTE standard was used for disease diagnosis, one of DINP’s metabolites, MCiOP, could potentially have an adverse effect on the occurrence of NAFLD (OR = 4.22, 95% CI = 1.10–16.2, *p* = 0.044). The reason for these differences could be partly due to the HSI and US.FLI diagnostic criteria relying on serum laboratory markers, which are influenced by various factors and are not as sensitive as VCTE. VCTE can directly measure liver stiffness, which more accurately reflects the degree of fibrosis associated with NAFLD (34). Another factor could be the differences in inclusion and exclusion criteria between studies, as well as the covariates considered, leading to different logistic regression models being constructed.

DINP emerges as a leading alternative in industrial applications to DEHP (35). Humans are widely exposed to DINP and May affect NAFLD through multiple pathways (36, 37). At present, the understanding of how DINP influences NAFLD is limited. Nonetheless, potential pathways through which DINP May contribute to NAFLD include alterations in lipid metabolism, oxidative stress, inflammation, and cellular communication mechanisms. Firstly, DINP promotes adipocyte differentiation and lipid accumulation in 3 T3-L1 pre-adipocytes through the activation of PPARγ (Peroxisome Proliferator-Activated Receptor Gamma), highlighting its role in adipogenesis and lipid metabolism (38). High doses of DINP can cause lipid metabolism disorders in male mice (39), suggesting that it May be conducive to the formation of fatty liver. Moreover, DINP modulates lipid metabolism by influencing the Endocannabinoid System (ECS), resulting in heightened accumulation of lipids and triglycerides in hepatic tissue (40). Secondly, DINP acts as a toxic agent that causes damage to liver tissues, induces oxidative stress by increasing ROS levels and depleting glutathione (GSH), promotes lipoperoxidation and DNA damage, and elevates pro-inflammatory cytokine levels (41). Moreover, DIDP can also lead to increased serum concentrations of ALT and AST among Balb/c mice, illustrating its potential to induce liver damage (42). Thirdly, the perturbation in ROS homeostasis, induced by DINP, catalyzes the activation of inflammatory responses, precipitating the secretion of inflammatory mediators. Studies have demonstrated that DINP induces an upregulation of IL-1 and TNF-*α* in Kunming mice (41). This phenomenon is ostensibly linked to the activation of the NF-κB signaling pathway, suggesting a mechanistic pathway through which DINP exerts its pro-inflammatory effects (43). Additionally, dermal exposure DINP enhances pulmonary inflammation via activation of the IL-31/TRPV1 pathway, leading to increased levels of IL-4, IL-5, IL-6, and IL-13, and decreased IFN-gamma in mice (44). DINP can also promote airway inflammation by activating the PI3K/AKT/NF-κB signaling pathway (45). Whether this mechanism exists in the liver remains to be further studied. Finally, DINP activates the MAPK-Erk1/2 pathway and, upon extended exposure, can inhibit gap junctional intercellular communication (GJIC), associated with the progression of fatty liver disease, fibrosis, and cirrhosis (46, 47). Regrettably, there is still a lack of research on human liver cells or tissues, and the mechanisms mentioned May exhibit interspecies variations, requiring further verification. However, it can still be stated that DINP May become a significant underlying factor influencing NAFLD through pathways such as lipid metabolism, oxidative stress, inflammatory responses, and cellular communication.

This study’s limitations are noteworthy. Initially, its cross-sectional approach, based on single-point phthalate level measurements from NHANES data, fails to capture the fluctuations in phthalate exposure. Phthalate metabolites have a short half-life in the body. This means that a single urine sample May only reflect short-term exposure (from a few hours to a few days) and May not accurately represent long-term or cumulative exposure. While a single measurement is still valuable for providing insights into recent exposure and possible associations with health outcomes. Future research should consider employing repeated sampling to obtain a more comprehensive understanding of long-term exposure. Subsequently, in exploring the association between multiple phthalates and NAFLD, this study leverages diverse diagnostic criteria, including HSI, US.FLI, and VCTE, through rigorous participant selection criteria. However, the availability of VCTE data is currently restricted to the 2017–2018 NHANES cycle. The anticipated update of the NHANES database will provide an opportunity for statistical analyses encompassing a broader dataset. Furthermore, although these three diagnostic methods can assess NAFLD, it is important to note that NAFLD progresses through multiple stages, such as Non-Alcoholic Fatty Liver (NAFL), Non-Alcoholic Steatohepatitis (NASH), fibrosis, and cirrhosis. Unfortunately, the current database does not provide sufficient data to allow for a stratified analysis of these stages. We acknowledge this limitation and recognize the need for future research to conduct more detailed studies on the staging of NAFLD to further investigate the association between phthalates and different stages of the disease. In addition, the covariates included in this study, such as smoking status, recreational physical activity, and insulin injection status, were all obtained through questionnaires, which May introduce recall bias. However, it is worth noting that the questionnaire data included in the NHANES database were collected by trained interviewers using standardized questionnaires and interview procedures, ensuring the scientific rigor and reliability of the data. Lastly, HSI, US.FLI, and VCTE are important tools in NAFLD research but are not considered gold standards like liver biopsy, which, despite its accuracy, is invasive and unsuitable for large-scale screenings. These non-invasive methods face limitations in sensitivity and specificity, potentially exaggerating phthalates’ role in NAFLD. However, these methods are acknowledged and have demonstrated utility in clinical diagnosis (26–28). Our study not only confirms a correlation between phthalates and NAFLD but also reveals that the choice of predictive criteria affects this correlation. VCTE is identified as a more accurate method than HSI and US.FLI for assessing liver stiffness (34), and its use has become prevalent for evaluating NAFLD. Our data suggest a notable link between DINP and NAFLD when diagnoses are made using VCTE criteria, which could improve early NAFLD detection and recommend that patients limit their exposure to DINP-containing plastic products to protect their liver health.

Numerous studies have investigated the association between phthalates and NAFLD. In our research, we consolidate existing viewpoints and uncovered the novel influence of different diagnostic methods on study outcomes. Additionally, we reveal that the correlation between DINP and NAFLD is significant, particularly when clinicians use non-invasive methods like the VCTE criteria for disease prediction. This awareness can prompt clinicians to consider environmental and chemical exposures as part of their assessment for NAFLD risk factors. Our study suggests that when utilizing VCTE criteria to assess NAFLD, the content of DINP secondary metabolites in urine May serve as a more potent predictor. It suggests that limiting exposure to DINP-containing products could be a viable preventative measure for at-risk populations. Furthermore, these insights advocate for incorporating routine environmental health screenings into clinical practice, enabling early intervention and personalized patient education. Future research should continue exploring the mechanistic pathways of DINP and validate these findings across diverse populations and longer-term studies.

## 5 Conclusion

This study establishes a positive link between phthalate exposure, specifically DINP (the parent body of the MCOP), and NAFLD risk in adults, highlighting the influence of diagnostic criteria on this relationship. Given DINP’s potential liver damage risk, it is imperative to conduct future case–control or cohort studies to confirm a causal relationship. Additionally, combining two or more 2-year cycles of the continuous NHANES is beneficial to understand this association better.

## Data Availability

The original contributions presented in the study are included in the article/Supplementary material, further inquiries can be directed to the corresponding author.

## Footnotes

^1^ noharm-europe.org

^2^ https://wwwn.cdc.gov/nchs/data/nhanes/2017-2018/labmethods/PHTHTE-J-MET-508.pdf

^3^ https://www.xsmartanalysis.com/beckman/login
