# Supplementary figures and images for: Association between phthalates exposure and non-alcoholic fatty liver disease under different diagnostic criteria: a cross-sectional study based on NHANES 2017 to 2018
Source: Front Public Health. 2024 Sep 25;12:1407976. doi: 10.3389/fpubh.2024.1407976 (PMC11462993; doi:10.3389/fpubh.2024.1407976)

**Figure S1. Parent Compounds of Phthalates and their Major Metabolites.**

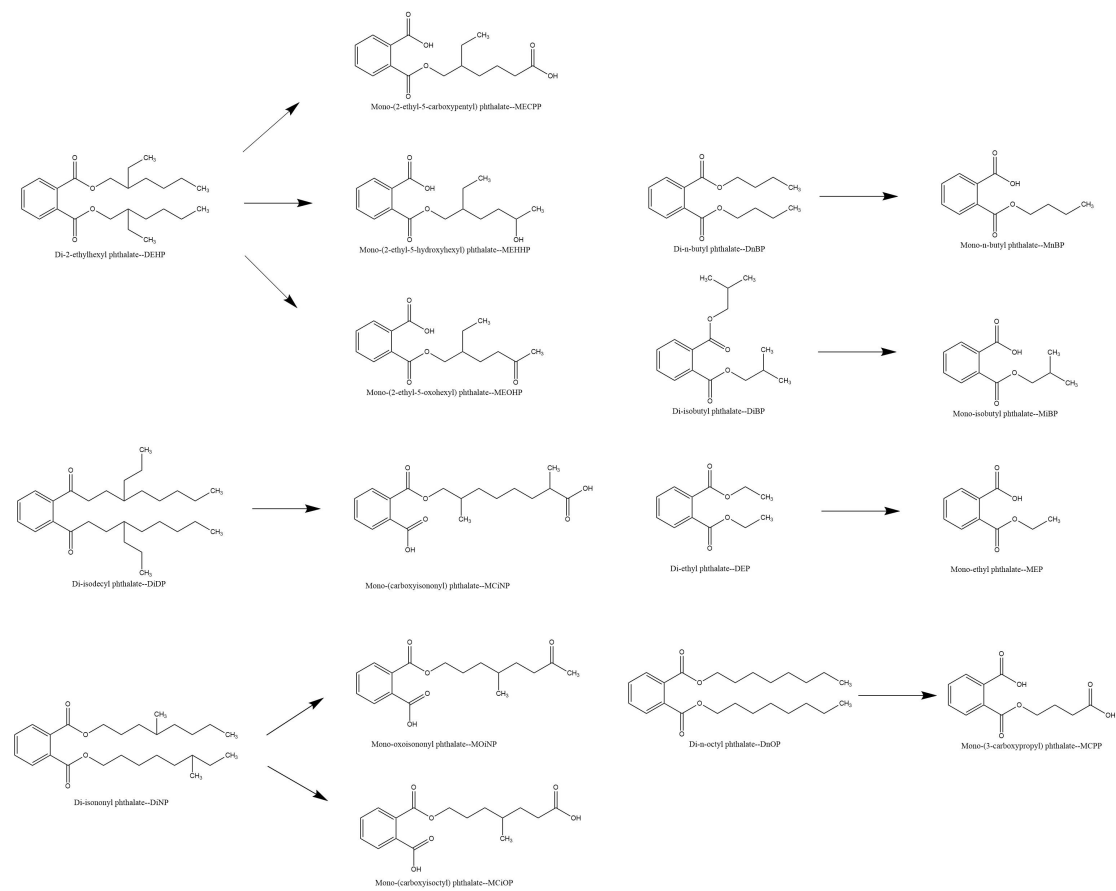

Supplement: Supplementary file 1 [file Image_1.pdf]
