# Supplementary material for: Association between phthalates exposure and non-alcoholic fatty liver disease under different diagnostic criteria: a cross-sectional study based on NHANES 2017 to 2018
Source: Front Public Health. 2024 Sep 25;12:1407976. doi: 10.3389/fpubh.2024.1407976 (PMC11462993; doi:10.3389/fpubh.2024.1407976)
Supplement: Supplementary file 2 [file Image_2.pdf]

Figure S2. Correlations between various indicators, those that have a significant correlation with NAFLD.

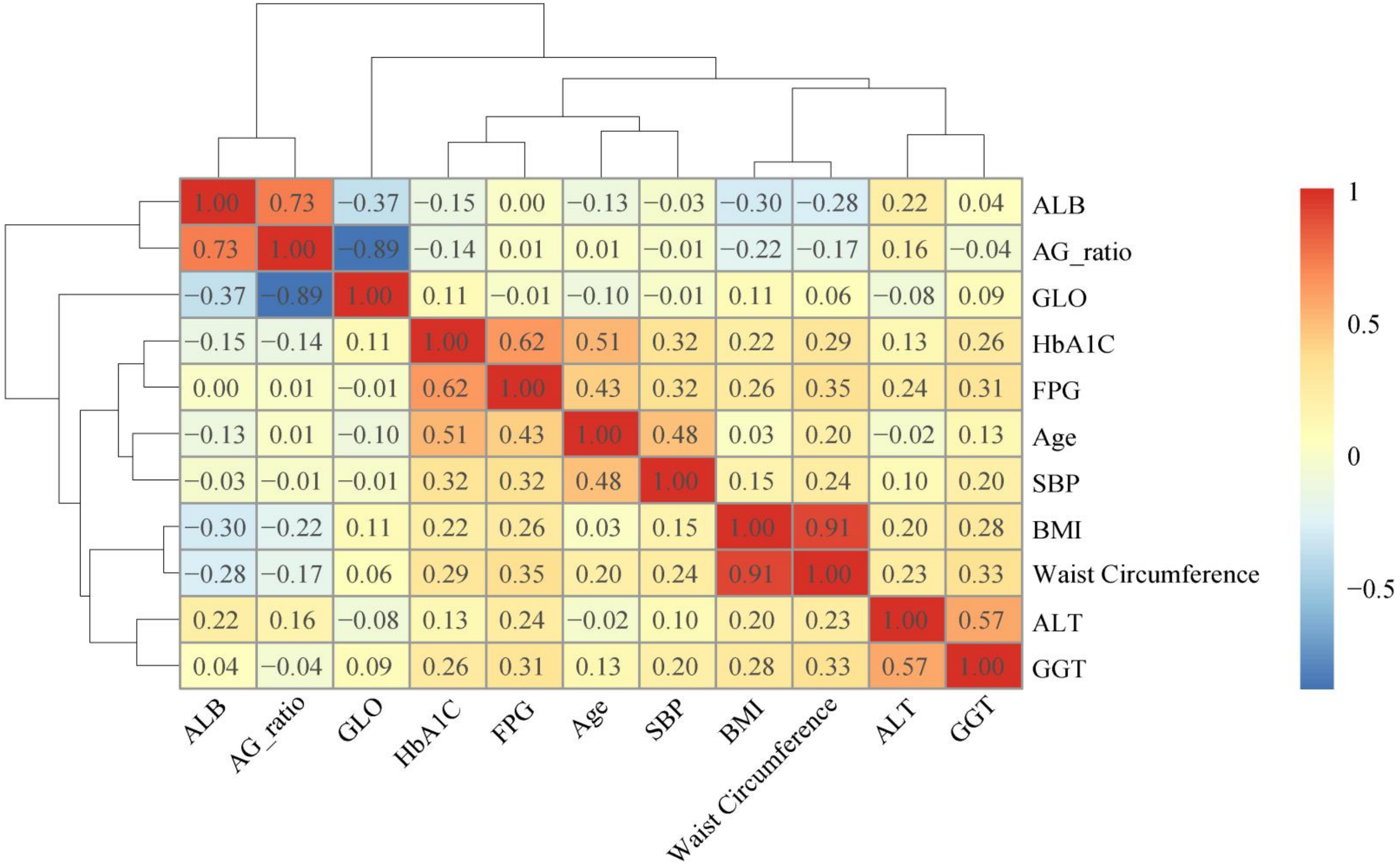

BMI,body mass index; SBP,systolic blood pressure; FPG,fasting plasma glucose; ALT,alanine aminotransferase; GGT,gamma-glutamyl transpeptidase; ALB,albumin; GLO,globulin; AG_ratio is a calculated value: albumin(g/dL)/globulin(g/dL);HbA1C, Glycated Hemoglobin. The numbers in the figure represent the correlation coefficient.
