# Supplementary material for: Association between phthalates exposure and non-alcoholic fatty liver disease under different diagnostic criteria: a cross-sectional study based on NHANES 2017 to 2018
Source: Front Public Health. 2024 Sep 25;12:1407976. doi: 10.3389/fpubh.2024.1407976 (PMC11462993; doi:10.3389/fpubh.2024.1407976)
Supplement: Supplementary file 3 [file Image_3.pdf]

**Figure S3.** ROC analysis to determine the relationship between NAFLD and two indicators: BMI and Waist Circumference.

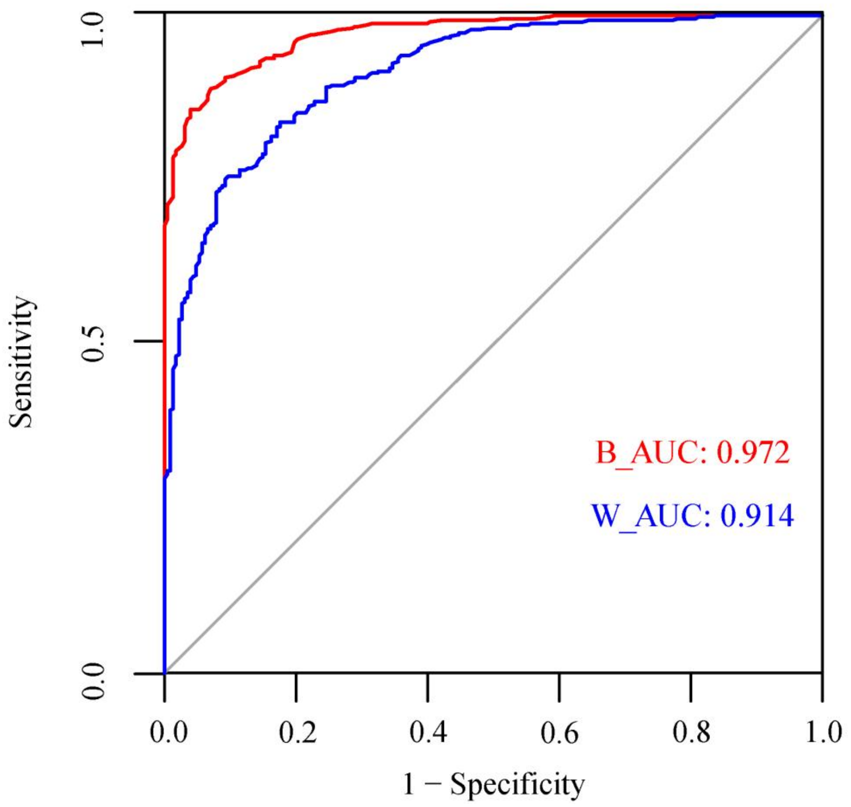


B, BMI which means body mass index; W, waist Circumference; AUC, area under the curve.
