# Supplementary material for: Association between phthalates exposure and non-alcoholic fatty liver disease under different diagnostic criteria: a cross-sectional study based on NHANES 2017 to 2018
Source: Front Public Health. 2024 Sep 25;12:1407976. doi: 10.3389/fpubh.2024.1407976 (PMC11462993; doi:10.3389/fpubh.2024.1407976)
Supplement: Supplementary file 4 [file Table_1.pdf]

Supplementary Material

Table S1. Parent Compounds of Phthalates and their Major Metabolites.

Parent phthalates, full name	Abb.	Major Metabolites, full name	Abb.	References*
Di-2-ethylhexyl phthalate	DEHP	Mono-2-ethyl-5-carboxypentyl phthalate	MECPP	
		Mono-(2-ethyl-5-hydroxyhexyl) phthalate	MEHHP	16234408
		Mono-(2-ethyl-5-oxohexyl) phthalate	MEOHP	
Replacements of DEHP				
Di-isodecyl phthalate	DIDP	Mono-(carboxyisononyl) phthalate	MCiNP	20870567
Di-isononyl phthalate	DINP	Mono-oxoisononyl phthalate	MOiNP	33125036/
		Mono-(carboxyisooctyl) phthalate	MCiOP	16882519
Di-n-butyl phthalate	DnBP	Mono-n-butyl phthalate	MnBP	19528056
Di-isobutyl phthalate	DIBP	Mono-isobutyl phthalate	MiBP	17604388
Di-ethyl phthalate	DEP	Mono-ethyl phthalate	MEP	19528056
Di-n-octyl phthalate	DnOP	Mono-(3-carboxypropyl) phthalate	MCPP	15840426

Abb.: abbreviation. *PMID is used here to indicate the source of the reference.
