# Supplementary material for: Association between phthalates exposure and non-alcoholic fatty liver disease under different diagnostic criteria: a cross-sectional study based on NHANES 2017 to 2018
Source: Front Public Health. 2024 Sep 25;12:1407976. doi: 10.3389/fpubh.2024.1407976 (PMC11462993; doi:10.3389/fpubh.2024.1407976)
Supplement: Supplementary file 5 [file Table_2.pdf]

Table S2. Model 2---Multivariate logistic regression analysis for NAFLD according to urinary phthalates levels in the male.

	HSI			US.FLI			VCTE		
**Characteristic**	**OR**[1]	**95% CI**[1]	***p-value***	**OR**[1]	**95% CI**[1]	***p-value***	**OR**[1]	**95% CI**[1]	***p-value***
**MEP**									
Q1	Ref.	Ref.		Ref.	Ref.		Ref.	Ref.	
Q2	2.52	0.23, 27.7	0.2	1.70	0.13, 21.6	0.5	5.43	0.15, 193	0.2
Q3	2.52	0.35, 18.2	0.2	1.85	0.01, 254	0.6	5.56	0.20, 156	0.2
Q4	0.80	0.05, 14.1	0.8	1.15	0.03, 44.7	0.9	8.23	0.24, 280	0.12
**MBP**									
Q1	Ref.	Ref.		Ref.	Ref.		Ref.	Ref.	
Q2	0.75	0.10, 5.41	0.6	1.29	0.05, 36.3	0.8	6.21	0.20, 196	0.2
Q3	2.09	0.09, 47.2	0.4	5.26	0.05, 611	0.3	6.67	0.17, 267	0.2
Q4	0.90	0.11, 7.35	0.9	1.71	0.06, 47.2	0.6	4.83	0.31, 75.5	0.13
**MiBP**									
Q1	Ref.	Ref.		Ref.	Ref.		Ref.	Ref.	
Q2	0.77	0.09, 6.68	0.7	1.10	0.09, 13.5	0.9	2.92	0.16, 53.4	0.3

	HSI			US.FLI			VCTE		
**Characteristic**	**OR**[1]	**95% CI**[1]	***p-value***	**OR**[1]	**95% CI**[1]	***p-value***	**OR**[1]	**95% CI**[1]	***p-value***
Q3	3.77	0.32, 43.9	0.15	4.34	0.28, 68.0	0.15	10.6	0.16, 697	0.14
Q4	0.57	0.02, 14.9	0.5	1.68	0.02, 115	0.6	3.18	0.44, 23.1	0.13
**MCPP**									
Q1	Ref.	Ref.		Ref.	Ref.		Ref.	Ref.	
Q2	0.74	0.05, 12.0	0.7	1.00	0.15, 6.86	>0.9	7.35	0.60, 90.5	0.076
Q3	1.56	0.09, 28.3	0.6	2.16	0.02, 201	0.5	2.80	0.20, 38.6	0.2
Q4	1.34	0.06, 32.0	0.7	1.38	0.05, 42.2	0.7	7.75	0.72, 83.1	0.066
**MONP**									
Q1	Ref.	Ref.		Ref.	Ref.		Ref.	Ref.	
Q2	2.94	0.04, 205	0.4	1.55	0.08, 30.9	0.6	2.09	0.09, 47.1	0.4
Q3	1.89	0.18, 20.3	0.4	1.04	0.05, 21.0	>0.9	1.21	0.05, 30.7	0.8
Q4	1.23	0.05, 27.5	0.8	0.56	0.02, 17.4	0.5	2.08	0.18, 23.7	0.3
**MEOHP**									
Q1	Ref.	Ref.		Ref.	Ref.		Ref.	Ref.	

		HSI			US.FLI			VCTE	
Characteristic	OR[1]	95% CI[1]	*p-value*	OR[1]	95% CI[1]	*p-value*	OR[1]	95% CI[1]	*p-value*
Q2	0.59	0.04, 8.66	0.5	1.56	0.15, 15.7	0.5	2.27	0.06, 86.0	0.4
Q3	1.44	0.11, 18.5	0.6	2.90	0.07, 115	0.3	2.44	0.19, 31.4	0.3
Q4	2.15	0.26, 17.7	0.3	2.31	0.03, 209	0.5	7.44	0.40, 138	0.10
**MEHHP**									
Q1	Ref.	Ref.		Ref.	Ref.		Ref.	Ref.	
Q2	0.37	0.02, 6.58	0.3	1.40	0.16, 12.0	0.6	0.84	0.03, 23.4	0.8
Q3	2.00	0.23, 17.0	0.3	4.54	0.41, 50.6	0.11	3.51	0.29, 42.1	0.2
Q4	1.84	0.19, 17.4	0.4	3.30	0.04, 244	0.4	5.25	0.25, 111	0.14
**MECPP**									
Q1	Ref.	Ref.		Ref.	Ref.		Ref.	Ref.	
Q2	0.46	0.02, 9.70	0.4	1.49	0.08, 27.3	0.6	0.83	0.04, 18.9	0.8
Q3	4.85	0.39, 60.6	0.11	8.72	0.15, 493	0.15	4.57	0.10, 216	0.2
Q4	0.85	0.04, 20.6	0.9	1.37	0.02, 83.8	0.8	5.03	0.39, 64.1	0.11
**MCOP**									

	HSI			US.FLI			VCTE		
**Characteristic**	**OR**[1]	**95% CI**[1]	***p-value***	**OR**[1]	**95% CI**[1]	***p-value***	**OR**[1]	**95% CI**[1]	***p-value***
Q1	Ref.	Ref.		Ref.	Ref.		Ref.	Ref.	
Q2	2.03	0.17, 25.0	0.3	1.58	0.04, 62.9	0.6	3.53	0.17, 73.8	0.2
Q3	1.10	0.11, 10.6	0.9	1.83	0.03, 134	0.6	3.76	0.27, 52.3	0.2
Q4	1.31	0.08, 21.4	0.7	1.36	0.04, 43.4	0.7	3.08	0.17, 57.0	0.2
**MCNP**									
Q1	Ref.	Ref.		Ref.	Ref.		Ref.	Ref.	
Q2	1.75	0.12, 26.1	0.5	0.94	0.02, 37.2	>0.9	1.02	0.06, 17.9	>0.9
Q3	1.96	0.17, 22.1	0.4	1.38	0.02, 111	0.8	2.62	0.15, 46.1	0.3
Q4	2.00	0.41, 9.75	0.2	3.21	0.11, 97.5	0.3	4.38	0.07, 281	0.3

1OR = Odds Ratio, CI = Confidence Interval;

MEP,Mono-ethyl phthalate; MBP,Mono-n-butyl phthalate; MiBP,Mono-isobutyl phthalate; MCPP,Mono-(3-carboxypropyl) phthalate; MONP,Mono-oxoisononyl phthalate; MEOHP,Mono-(2-ethyl-5-oxohexyl) phthalate; MEHHP,Mono-(2-ethyl-5-hydroxyhexyl) phthalate; MECPP,Mono-2-ethyl-5-carboxypentyl phthalate; MCOP,Mono(carboxyoctyl) Phthalate; MCNP,Mono(carboxynonyl) Phthalate;
