# Supplementary material for: Association between phthalates exposure and non-alcoholic fatty liver disease under different diagnostic criteria: a cross-sectional study based on NHANES 2017 to 2018
Source: Front Public Health. 2024 Sep 25;12:1407976. doi: 10.3389/fpubh.2024.1407976 (PMC11462993; doi:10.3389/fpubh.2024.1407976)
Supplement: Supplementary file 6 [file Table_3.pdf]

Table S3. Model 2---Multivariate logistic regression analysis for NAFLD according to urinary phthalates levels in the female.

	HSI			US.FLI			VCTE		
**Characteristic**	**OR[1]**	**95% CI[1]**	***p-value***	**OR[1]**	**95% CI[1]**	***p-value***	**OR[1]**	**95% CI[1]**	***p-value***
**MEP**									
Q1	Ref.	Ref.		Ref.	Ref.		Ref.	Ref.	
Q2	4.93	0.16, 150	0.2	39.3	0.13, 11,456	0.11	5.40	0.27, 108	0.14
Q3	3.47	0.43, 28.2	0.13	27.0	0.67, 1,088	0.062	1.36	0.08, 23.2	0.7
Q4	2.04	0.06, 73.1	0.5	20.5	0.22, 1,884	0.10	1.50	0.07, 32.6	0.6
**MBP**									
Q1	Ref.	Ref.		Ref.	Ref.		Ref.	Ref.	
Q2	0.95	0.07, 13.7	>0.9	3.19	0.08, 133	0.3	0.20	0.00, 15.6	0.3
Q3	4.08	0.32, 51.8	0.14	11.1	0.36, 344	0.095	1.87	0.20, 17.9	0.4
Q4	2.40	0.07, 80.2	0.4	8.79	0.15, 502	0.15	0.50	0.01, 22.8	0.5
**MiBP**									
Q1	Ref.	Ref.		Ref.	Ref.		Ref.	Ref.	
Q2	2.82	0.19, 42.4	0.2	6.86	0.49, 96.9	0.089	0.77	0.08, 7.34	0.7

	HSI			US.FLI			VCTE		
**Characteristic**	**OR**[1]	**95% CI**[1]	***p-value***	**OR**[1]	**95% CI**[1]	***p-value***	**OR**[1]	**95% CI**[1]	***p-value***
Q3	2.66	0.04, 188	0.4	11.2	0.18, 692	0.13	0.91	0.03, 28.3	>0.9
Q4	4.96	0.15, 163	0.2	29.2	0.96, 887	0.051	1.29	0.10, 17.2	0.7
**MCPP**									
Q1	Ref.	Ref.		Ref.	Ref.		Ref.	Ref.	
Q2	2.07	0.15, 28.2	0.4	4.30	0.16, 117	0.2	0.20	0.01, 4.60	0.2
Q3	7.73	0.20, 304	0.14	13.0	0.31, 543	0.10	1.88	0.15, 23.8	0.4
Q4	2.00	0.09, 45.5	0.4	6.79	0.14, 318	0.2	0.49	0.03, 9.06	0.4
**MONP**									
Q1	Ref.	Ref.		Ref.	Ref.		Ref.	Ref.	
Q2	4.40	0.26, 74.4	0.2	0.77	0.01, 61.4	0.8	1.44	0.13, 15.9	0.6
Q3	4.66	0.12, 179	0.2	3.49	0.10, 127	0.3	3.42	0.23, 51.2	0.2
Q4	2.22	0.13, 37.4	0.3	2.62	0.32, 21.2	0.2	1.02	0.21, 5.10	>0.9
**MEOHP**									
Q1	Ref.	Ref.		Ref.	Ref.		Ref.	Ref.	

	HSI			US.FLI			VCTE		
**Characteristic**	**OR**[1]	**95% CI**[1]	***p-value***	**OR**[1]	**95% CI**[1]	***p-value***	**OR**[1]	**95% CI**[1]	***p-value***
Q2	1.68	0.10, 27.6	0.5	3.59	0.11, 122	0.3	0.57	0.01, 22.0	0.6
Q3	5.29	0.18, 159	0.2	3.01	0.05, 186	0.4	5.19	0.15, 175	0.2
Q4	2.11	0.55, 8.03	0.14	4.05	0.39, 41.5	0.12	1.50	0.13, 17.8	0.6
**MEHHP**									
Q1	Ref.	Ref.		Ref.	Ref.		Ref.	Ref.	
Q2	3.93	0.08, 190	0.3	2.19	0.04, 133	0.5	0.65	0.04, 10.4	0.6
Q3	5.13	0.16, 164	0.2	1.21	0.01, 165	0.9	6.62	0.32, 137	0.12
Q4	5.38	0.48, 60.5	0.10	4.76	0.45, 50.1	0.10	2.40	0.28, 20.9	0.2
**MECPP**									
Q1	Ref.	Ref.		Ref.	Ref.		Ref.	Ref.	
Q2	1.63	0.07, 36.2	0.6	0.34	0.00, 29.0	0.4	1.85	0.19, 17.9	0.4
Q3	7.60	0.09, 649	0.2	2.28	0.07, 76.6	0.4	4.26	0.17, 107	0.2
Q4	3.09	0.34, 27.8	0.2	2.19	0.29, 16.3	0.2	2.96	0.30, 28.8	0.2
**MCOP**									

	HSI			US.FLI			VCTE		
**Characteristic**	**OR[1]**	**95% CI[1]**	***p-value***	**OR[1]**	**95% CI[1]**	***p-value***	**OR[1]**	**95% CI[1]**	***p-value***
Q1	Ref.	Ref.		Ref.	Ref.		Ref.	Ref.	
Q2	2.69	0.14, 53.0	0.3	1.00	0.04, 24.1	>0.9	1.15	0.09, 15.1	0.8
Q3	3.28	0.16, 66.1	0.2	1.86	0.10, 35.2	0.5	4.49	0.59, 34.2	0.086
Q4	4.87	0.19, 124	0.2	5.35	0.26, 110	0.14	3.17	0.52, 19.3	0.11
**MCNP**									
Q1	Ref.	Ref.		Ref.	Ref.		Ref.	Ref.	
Q2	3.23	0.26, 39.4	0.2	2.85	0.07, 122	0.4	0.77	0.03, 18.3	0.8
Q3	6.18	0.28, 137	0.13	11.3	0.18, 715	0.13	2.89	0.14, 59.8	0.3
Q4	2.65	0.20, 35.0	0.2	7.57	0.24, 239	0.13	1.97	0.09, 43.2	0.4

1OR = Odds Ratio, CI = Confidence Interval;

MEP,Mono-ethyl phthalate; MBP,Mono-n-butyl phthalate; MiBP,Mono-isobutyl phthalate; MCPP,Mono-(3-carboxypropyl) phthalate; MONP,Mono-oxoisononyl phthalate; MEOHP,Mono-(2-ethyl-5-oxohexyl) phthalate; MEHHP,Mono-(2-ethyl-5-hydroxyhexyl) phthalate; MECPP,Mono-2-ethyl-5-carboxypentyl phthalate; MCOP,Mono(carboxyoctyl) Phthalate; MCNP,Mono(carboxynonyl) Phthalate;
